# Identification of commensal gut microbiota signatures as predictors of clinical severity and disease progression in multiple sclerosis

**DOI:** 10.1038/s41598-024-64369-x

**Published:** 2024-07-03

**Authors:** Theresa L. Montgomery, Qin Wang, Ali Mirza, Deanna Dwyer, Qi Wu, Catherine A. Dowling, Jacob W. S. Martens, Jennifer Yang, Dimitry N. Krementsov, Yang Mao-Draayer

**Affiliations:** 1 Department of Biomedical and Health Sciences, University of Vermont, Burlington, VT 05401 USA; 2 Autoimmunity Center of Excellence, University of Michigan Medical School, Ann Arbor, MI 48109 USA; 3 Pharmacoepidemiology in Multiple Sclerosis Research Group, The University of British Columbia, Vancouver, BC V6T 2B5 Canada; 4 Autoimmunity Center of Excellence, Multiple Sclerosis Center of Excellence, Arthritis and Clinical Immunology Research Program, Oklahoma Medical Research Foundation, Oklahoma City, OK 73104 USA

**Keywords:** Multiple sclerosis, Vitamin K, Progression, Akkermansia, Gut microbiota, Bacterial metabolism, Predictive markers, Multiple sclerosis, Computational biology and bioinformatics, Microbiome

## Abstract

Multiple sclerosis (MS) is a chronic autoimmune disease of the central nervous system and a leading cause of neurological disability in young adults. Clinical presentation and disease course are highly heterogeneous. Typically, disease progression occurs over time and is characterized by the gradual accumulation of disability. The risk of developing MS is driven by complex interactions between genetic and environmental factors, including the gut microbiome. How the commensal gut microbiota impacts disease severity and progression over time remains unknown. In a longitudinal study, disability status and associated clinical features in 58 MS patients were tracked over 4.2 ± 0.98 years, and the baseline fecal gut microbiome was characterized via 16S amplicon sequencing. Progressor status, defined as patients with an increase in Expanded Disability Status Scale (EDSS), were correlated with features of the gut microbiome to determine candidate microbiota associated with risk of MS disease progression. We found no overt differences in microbial community diversity and overall structure between MS patients exhibiting disease progression and non-progressors. However, a total of 41 bacterial species were associated with worsening disease, including a marked depletion in *Akkermansia*, *Lachnospiraceae,* and *Oscillospiraceae*, with an expansion of *Alloprevotella*, *Prevotella-9*, and *Rhodospirillales*. Analysis of the metabolic potential of the inferred metagenome from taxa associated with progression revealed enrichment in oxidative stress-inducing aerobic respiration at the expense of microbial vitamin K_2_ production (linked to *Akkermansia*), and a depletion in SCFA metabolism (linked to *Oscillospiraceae*). Further, as a proof of principle, statistical modeling demonstrated that microbiota composition and clinical features were sufficient to predict disease progression. Additionally, we found that constipation, a frequent gastrointestinal comorbidity among MS patients, exhibited a divergent microbial signature compared with progressor status. These results demonstrate a proof of principle for the utility of the gut microbiome for predicting disease progression in MS in a small well-defined cohort. Further, analysis of the inferred metagenome suggested that oxidative stress, vitamin K_2_, and SCFAs are associated with progression, warranting future functional validation and mechanistic study.

## Introduction

Multiple sclerosis (MS) is a chronic demyelinating autoimmune disease of the central nervous system (CNS) that predominantly affects young adults^1–3^. However, the clinical presentation, onset, and disease course of MS may vary depending on genetic susceptibility and environmental factors, including the gut microbiome^4–7^. The severity and progression of MS is heterogeneous and varies from person to person^8^. Over time most cases of relapsing–remitting MS (RRMS) progressively worsen in severity and transition to secondary progressive MS (SPMS) with accumulation of disability^9^. Approximately 5–10% of patients experience only mild relapse without significant disease progression. These patients, classified as non-progressive benign MS (BMS/NPMS), never suffer from significant disabilities^10–12^. Prior studies that have analyzed SPMS typically compare them to RRMS which is a poor comparator because patients with RRMS are usually younger, currently taking a disease modifying therapy (DMT), have shorter disease duration, and are heterogeneous in terms of the trajectory toward SPMS. BMS is distinct from SPMS in that it lacks disease progression. Since patients with BMS have a similar disease duration to SPMS and differ in the rate of progression, they serve as an ideal comparator to identify mechanisms underlying MS progression. Using the novel comparison to BMS, we uncovered dysregulation of B cell differentiation and function, humoral immunity, iron homeostasis, and lipid metabolism is associated with MS disease progression^13^.

In recent years, the treatment paradigm for MS has shifted towards early aggressive intervention to prevent disease progression and irreversible CNS damage. Given that not all MS patients progress in their disease, there is an unmet need to identify patients who are at high risk for progression and require more aggressive treatment strategies to prevent long-term disability. Our prior studies also identified candidate plasma biomarkers capable of distinguishing different progressive stages of MS^14^. Here, we examine the potential of using the gut microbiome as a prognostic approach to assess risk of progression.

Prior animal studies have shown that the gut microbiome can modulate peripheral and central nervous system immunity^15,16^. Specifically, microbiota can alter demyelination and the permeability of the blood–brain barrier (BBB), which suggests potential as a predictive factor for relapse risk in MS^17–20^. Previous studies have also shown that MS patients have a distinct gut microbiome as compared to healthy patients, albeit with significant cohort to cohort variation, with the most consistent finding being increased abundance of *Akkermansia muciniphila* (*A. muciniphila*) in MS patients^19,21,22^. Functionally, several studies have suggested MS patient gut microbiota has a diminished capacity to produce short-chain fatty acids (SCFAs), which have a known role in limiting the production of pro-inflammatory cytokines^18,23–28^ and in enhancing the production of anti-inflammatory cytokines via an increase in regulatory T cells through SCFA G-protein-coupled receptors^29^. A decrease in abundance of SCFA-producing obligate anaerobes has been demonstrated in instances of increased oxygen-dependent aerobic respiration in the gut^30,31^. Further, a decrease in SCFAs in patients with SPMS has also been reported, suggesting possible utility as a marker for progression. Importantly, gastrointestinal symptoms are frequently reported in MS and often postulated to confound gut microbiome studies in these patients, especially in studies comparing healthy controls who typically lack constipation^32–36^, yet no studies have directly documented the effects of this confounder on gut microbiota in MS patients.

While cross-sectional studies of the gut microbiome in MS have revealed some differences from healthy controls and associations with current disease activity^19,28,37–44^, longitudinal studies exploring gut microbiota composition and clinical measures in MS remain sparse. Further, available longitudinal studies mostly focus on the potential relationship between the gut microbiome and relapse activity. Thus, it remains unclear whether gut microbiota are associated with MS progression and disability worsening. We utilized longitudinal tracking of disease severity (via the change in Expanded Disability Status Scale (EDSS)score) and the fecal gut microbiome (via 16S rRNA gene sequencing) to elucidate any relationship with MS progression in a small well-defined cohort, as proof of principle. We took a novel approach comparing progressors vs. non-progressors across the spectrum of MS disease types. We found a total of 41 bacterial taxa were associated with worsening disease, including a depletion in *Akkermansia* and SCFA-producing *Lachnospiraceae* and *Oscillospiraceae* species, with an expansion of *Alloprevotella*, *Prevotella-9*, and *Rhodospirillales*. Pathway analysis on the inferred metagenome of taxa associated with progression revealed an enrichment in oxidative stress-inducing aerobic respiration at the expense of microbial vitamin K2 production, and a decrease in SCFA metabolism, warranting future functional validation. Our study showed that gut microbial signatures could also be used as potential predictors for disease progression across MS disease types. Gut microbiota aided in clustering of clinical features and identification of potential confounding effects of MS-associated symptoms; constipation was not a significant confounder when examining the gut microbiome in relationship to MS disease progression. Our results offer insights into pathological mechanisms unique to progression, including specific microbial pathways, metabolites, or interactions that could contribute to worsening disease, to be validated in larger cohorts in future prospective studies.

## Results

### Gut microbiome baseline diversity and community structure does not differ in MS patients that exhibit disease progression

**Figure 1:**
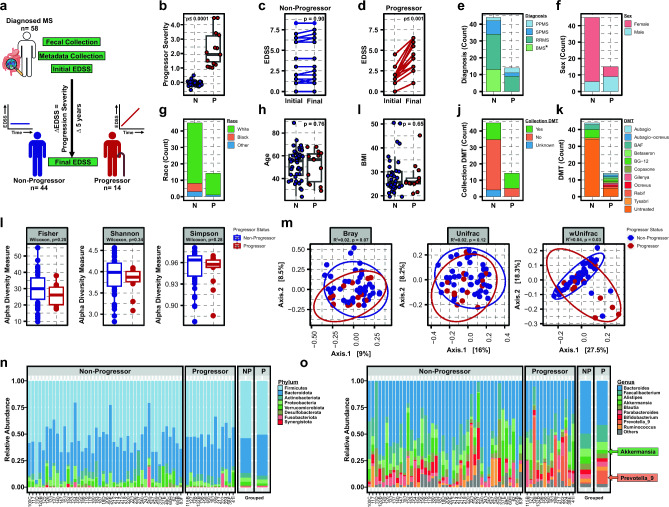
MS disease progression is not associated with differences in baseline gut microbiome alpha and beta diversity. (**a**) Schematic of clinical study design depicting participant binning strategy based on longitudinal disease progression where a non-progressor = change in EDSS of ≤0.5 and a progressor = change in >0.5. Bar graphs, line diagrams, and scatter plots of selected study metadata as outlined in Table 1, segregated by participant progressor status, including: (**b**) progressor severity (change in EDSS over study period), (**c**) non-progressor and (**d**) progressor initial and final EDSS, (**e**) MS-subtype, (**f**) sex (**g**) self-identified race, (**h**) age, (**i**) BMI, (**j**) collection DMT status (**k**) DMT prior history. p-values represent a two-tailed binomial test of significant deviation from expected outcome of a 50% division of between progressors and non-progressors for categorical data, or standard t-test for numerical data, with significance at p ≤ 0.05. Complete statistical analysis of subject metadata is included in Tables 1 and 2. (**l**) Alpha (Fisher, Shannon and Simpson) and (**m**) beta (Bray–Curtis dissimilarity, Unifrac, wUnifrac) diversity analysis in progressors and non-progressors, as analyzed using Wilcoxon rank sum non-parametric or Adonis testing, respectively. Stacked bar plots depicting microbiota composition at bacterial phylum (**n**) and top 10 most abundant genera (**o**), as relative proportion of total 16S V4 amplicon reads within each taxonomic rank.

Fecal samples were collected from 58 MS patients recruited from the University of Michigan Medical School to profile the gut microbiome. In addition, initial disability status was assessed, followed by longitudinal tracking of EDSS from baseline for an average 4.2 ± 0.98 years (Fig. 1A). All subjects completed a clinical survey reporting subject demographics, disease subtype and history, medications, and any comorbidities (Fig. 1A and Table 1). In total, 13 patients (22.4%) had benign MS (BMS), 30 (51.7%) relapsing–remitting MS (RRMS), 10 (17.2%) secondary-progressive MS (SPMS), and 5 (8.6%) were diagnosed with primary-progressive MS (PPMS) (Table 1). We intentionally enrolled a large number of BMS patients, given lack of published studies using this population. Our recent work by utilizing BMS as comparator has allowed for discovery of distinct transcriptomics, plasma soluble factors and lipid metabolic signatures associated with disease^13,14,45^. To test the hypothesis that the gut microbiome may be a factor for predicting disease progression in all MS patients, we grouped patients independent of MS-subtype, based instead on disease progression within the study time period. Specifically, progressor severity was calculated as the change in EDSS from baseline (final EDSS—initial EDSS). Subjects were stratified by progressor severity where a change in EDSS of > 0.5 was considered disease progression (Progressor (P), n = 14), while a change of ≤0.5 was considered no change in disease severity (Non-Progressor (NP), n = 44) (Fig. 1b and Table 1). While no differences in initial EDSS between non-progressors and progressors (mean NP = 2.2 and P = 1.9, p = 0.52) was observed, final EDSS was significantly different (mean NP = 2.3 and P = 4.2, p ≤ 0.01), reflected as a significant change in EDSS specifically associated with disease progression (p ≤ 0.001) (Fig. 1b–d and Table 1). Further, there was the expected difference in MS-subtype between progressor and non-progressors, including an increase in BMS patients as non-progressor (p ≤ 0.001) (Fig. 1e and Table 1), and a greater proportion of females in non-progressors (p ≤ 0.0001) (Fig. 1F and Table 1). Importantly, there was a modest but significant overrepresentation of White/Caucasian self-identified patients in progressors (p ≤ 0.01), while no difference in age, BMI, or DMT exposure were observed (Fig. 1g–k and Table 1). Further, DMT usage at the time of fecal collection was low overall, at 32.8%, driven by non-progressors, with a large BMS and/or otherwise stable disease sub-types not requiring therapeutic treatment.

**Table 1:** Demographics and clinical characteristics of study participants.

Reported n, (%)	Non-progressor n = 44	Progressor n = 14	Total n = 58	Statistical analysis
Diagnosis	BMS = 13 (29.5%) RRMS = 21 (47.7%) SPMS = 8 (18.2%) PPMS = 2 (4.5%)	BMS = 0 (0%) RRMS = 9 (64.3%) SPMS = 2 (14.3%) PPMS = 3 (21.4%)	BMS = 13 (22.4%) RRMS = 30 (51.7%) SPMS = 10 (17.2%) PPMS = 5 (8.6%)	BMS ≤ 0.001 RRMS = 0.04 SPMS = 0.11 PPMS = > 0.9999
Age at collection, years: average (SD; range)	51.6 (12.4; 28–74)	51.1 (14.0; 29–68)	51.5 (12.7; 28–74)	Age = 0.91
Sex	Male = 6 (13.6%) Female = 38 (86.4%)	Male = 9 (64.3%) Female = 5 (35.7%)	Male = 15 (25.9%) Female = 43 (74.1%)	Male = 0.61 Female ≤ 0.0001
Self-identified race	Other = 3 (6.8%) Black = 5 (11.4%) White = 36 (81.8%)	Other = 1 (7.1%) Black = 0 (0.0%) White = 13 (92.9%)	Other = 4 (6.9%) Black = 5 (8.6%) White = 49 (84.5%)	Other = 0.63 Black = 0.06 White < 0.01
Height, in average (SD; range)	65.0 (3.5; 58.5–76)	69.5 (4.1; 62.0–75.0)	66.2 (4.2; 58.5–76.0)	P ≤ 0.003
Weight, lbs. average (SD; range)	174.7 (54.8; 107–330)	188.2 (40.9; 137.5–280)	178.2 (51.5; 107–330)	P = 0.36
BMI: average (SD; range)	28.4 (7.7; 19.6–50.5)	27.4 (6.2; 20.9–45.2)	28.2 (7.3; 19.6–50.5)	P = 0.65
Age of onset, years: average (SD; range)	34.0 (10.6;17–59)	32.5 (9.0; 20–52)	33.6 (10.2; 17–59)	P = 0.61
Disease duration, years: average (SD; range)	17.8 (13.0; < 1–46)	18.6 (12.8; 2–42)	18.0 (12.9; < 1–46)	P = 0.83
Initial EDSS (SD; range)	2.2 (2.4; 0–9)	1.9 (1.0; 0–4.5)	2.1 (2.2; 0–9)	P = 0.52
Final EDSS	2.3 (2.5; 0–9)	4.2 (1.8; 1–6.5)	2.7 (2.5; 0–9)	P ≤ 0.01
Severity score final EDSS − Initial EDSS average (SD; range)	0.01 (0.13; -0.05–0.05)	2.23 (1.23; 1–4.5)	0.57 (1.2; -0.5–4.5)	P ≤ 0.0001
Disease-modifying therapy (DMT) at collection: no/yes	*BMS = 13 (29.5%), 0 (0.0%) RRMS = 15 (34.1%), 6 (13.6%) SPMS = 4 (9.1%), 4 (9.1%) PPMS = 2 (4.5%), 0 (0.0%)	BMS = 0 (0.0%), 0 (0.0%) RRMS = 3 (21.4%), 6 (42.9%) SPMS = 1 (7.14%), 1 (7.14%) PPMS = 1 (7.14%), 2 (14.3%)	*BMS = 13 (22.4%), 0 (0.0%) RRMS = 18 (31.0%), 12 (20.7%) SPMS = 5 (8.6%), 5 (8.6%) PPMS = 3 (5.2%), 2 (3.4%)	P > 0.9999*
Recent relapse (any)	6 (13.6%)	4 (28.6%)	10 (17.2%)	P = 0.75
Recent MRI activity (any)	9 (20.5%)	4 (28.6%)	13 (22.4%)	P = 0.27

*BMS specifically enrolled as non-progressors not requiring DMT usage; p-value denotes DMT usage "yes" between progressors and non-progressors.

To pinpoint gut microbial signatures that predispose individuals towards disease progression, we profiled the gut microbiome composition present in baseline fecal samples via 16S rRNA gene sequencing (as detailed in the Methods section). Briefly, DNA was extracted from fecal samples, followed by amplification of the V4 region of the 16S rRNA gene and sequenced using the Illumina MiSeq platform. Reads were trimmed and filtered to remove low quality or short reads, with standard error learning and merging parameters^46^. Following chimera removal (consensus method), an average of 168,020 reads per sample remained, with a 76.53% read retention. Amplicon sequence variant (ASV) level taxonomic assignment was performed using the SILVA database (version 138.1) and the established DADA2 (version 1.24.0) pipeline^46–49^ resulting in a total 3434 unique ASVs. Downstream data analysis and visualization primarily utilized *phyloseq* (version 1.40.0) with a detailed description of bioinformatic approaches provided in the Materials and Methods section^50^.

We first examined gut microbiome diversity between non-progressors and progressors. Consistent with previous studies comparing healthy controls to MS subjects, we found no overt difference in intra-group species richness as measured by alpha diversity (Fisher, Shannon, Simpson, Wilcoxon rank sum tests at p = 0.20, p = 0.34, and p = 0.28 respectively) (Fig. 1l)^37,51^. To examine differences in overall community structure, beta diversity was analyzed between disease progressors and non-progressors. We found no overt difference in community structure as measured by two of three metrics of beta diversity (Bray and Unifrac rank sum tests at p = 0.07, p = 0.12, respectively, and R^2^ = 0.02). While weighted Unifrac, which accounts for both presence and abundance of microbial taxa, passed FDR constraints, the impact on microbial diversity was minor (R^2^ = 0.04, p = 0.03) (Fig. 1m). Taken together, these data suggest that no major changes exist in species diversity within (alpha) or between (beta) subjects that do or do not progress in their disease course. To broadly visualize the composition of the gut microbiome between disease progressors and non-progressors, we examined the distribution of bacterial phyla and genera (Figs. 1n–o and S1). Bacterial phyla were comprised of the expected taxonomic constituents present within the human gut, including a prominent abundance of Firmicutes and Bacteroidota (Fig. 1n), while the top 10 most abundant genera included *Bacteroides, Faecalibacterium, Akkermansia, and Prevotella-9* (Fig. 1 o) among others.

**Figure 2:**
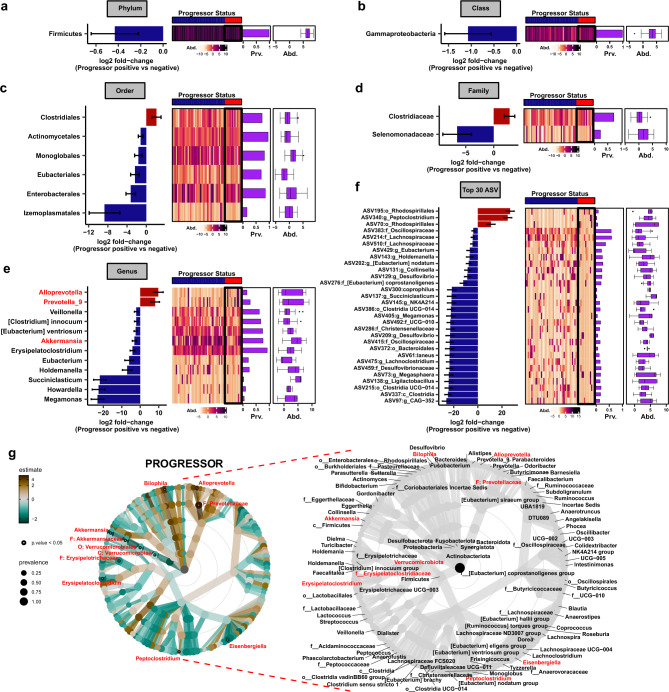
MS disease progressors exhibit a unique baseline gut microbial signature. Differentially abundant taxa between subjects with or without disease progression by (**a**) phylum, (**b**) class, (**c**) order, (**d**) family, (**e**) genus, and (**f**) top 30 ASVs represented as taxonomic best-hit, as determined by DESeq2 analysis, using a cutoff of p_adj_ ≤ 0.05. Log2 fold-change reflects increased abundance in progressors when positive, and decreased abundance when negative. Heatmaps and bar graphs of prevalence as well as center log ratio (CLR) transformed abundance graphs are aligned to the right. (**e**) Taxonomic association heat tree of differentially abundant microbiota determined by total sum scaling (TSS) log2 linear regression through the level of genus. Shown on the right is a phylogenetic key for the heat tree shown on the left. Data was filtered at a minimum prevalence of 0.1, significance determined at a cutoff of p_adj_ ≤ 0.05. Node size indicates proportional prevalence, significant nodes are represented as open circles (or red font in the key), warmer colors are enriched in progressors, with cooler colors indicating depletion.

To specifically assess significant changes in gut microbiome composition occurring at each taxonomic level between non-progressor and progressors, differential abundance analysis was performed using DESeq2 (version 1.36.0)^52^, using at p_adj_ ≤ 0.05 as the cutoff for differential abundance (Tables S1–S6). Consistent with exploratory visualization of taxonomic distributions in Fig. 1n, minimal phylum level alterations were observed, including a modest reduction in Firmicutes in disease progressors (Fig. 2a). At each lower taxonomic level, more taxa were found to have significantly lower abundance in the progressor group compared with non-progressors. A single class was depleted in progressors – *Gammaproteobacteria—*driven by the *Enterobacterales* order (Fig. 2b,c). Additional order level differences included depletion of *Eubacteriales* driven by a single ASV resolving to *Eubacterium* at the genus level, as well as a reduction in *Actinomycetales* and *Monoglobales* (Fig. 2c–f). Notably, the enrichment of the *Clostridiales* order in progressors was carried through to the taxonomic rank of family, with a marked increase in *Clostridiaceae* (Fig. 2c,d). Analysis of bacterial genera revealed an enrichment in *Prevotella-9* and *Alloprevotella*, with a marked depletion of *Holdemanella*, *Succiniclasticum*, *Howardella*, and *Megamonas,* all driven by the differential abundance of individual ASVs, as well as a significant reduction in *Akkermansia* (Fig. 2e,f). Given that ASVs often fail to resolve to the level of species, taxonomy was collapsed to the lowest taxonomic rank assigned and represented as a taxonomic best-hit for fold-change analysis of sequence variants. Differential abundance of ASVs revealed an increase in two *Rhodospirillales* ASVs, and a reduction in several *Lachnospiraceae*, *Desulfovibrio*, and *Oscillospiraceae* ASVs (Fig. 2f). As fold-change analysis does not account for phylogenetic relationships, differential abundance testing was also modeled using total sum scaling (TSS) log2 linear regression and represented as a taxonomic association heat tree (Fig. 2g and Table S7). Consistent with fold-change analysis, an increase in *Prevotellaceae* and *Alloprevotella* was observed as well as a marked depletion of *Akkermansia* (phylum V*errucomicrobiota*), as suggested by exploratory observations of the top 10 genera between progressors and non-progressors (Figs. 1o, 2g, and Table S7). Taken together, these data are indicative of a unique baseline gut microbial signature of MS disease progression that is not readily apparent through community structure and diversity analysis alone.

### Identification of microbiota signatures associated with disease progression

**Figure 3:**
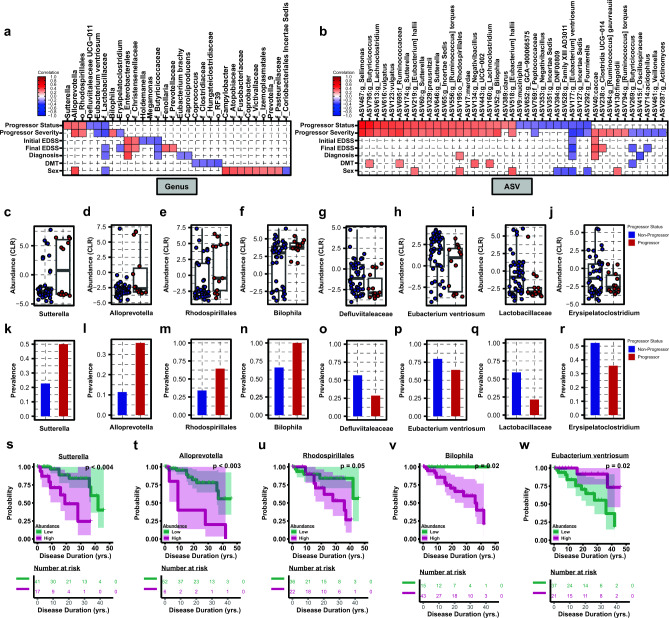
Abundance and prevalence of progressor-associated microbiota. Association of (**a**) genera and (**b**) ASVs with progressor status and closely related subject metadata, as determined by Spearman rank correlation ≥|0.2|, at p_adj_ ≤ 0.05. Taxa are sorted from high to low rho-value within each metadata group top to bottom on y-axes, where warmer colors are indicative of positive association (increased abundance) and cooler colors represent negative association (decreased abundance). Metadata binning strategy is listed in Table 1. CLR transformed abundance (**c**–**j**) and % prevalence at each taxonomic rank (**k**–**r**) are shown for each genera associated with progressor status. Kaplan–Meier curves predicting probability of disease progression over subject disease duration (yrs) using cohorts stratified based on abundance of (**s**) *Sutterella* (low/high), (**t**) *Alloprevotella* (low/high), (**u**) *Rhodospirillales* (low/high), (**v**) *Bilophila* (low/higher) and (**w**) *Eubacterium ventriosum* (low/high). Significance represents log-rank p-value ≥ 0.05 for differential probability of disease progression survival between microbiota driven strata.

To begin to assess candidate microbiota that most closely correlated specifically with disease progression, gut microbiota collapsed at each taxonomic rank was cross-correlated (Spearman rank correlation, cutoff at rho ≥|0.2|, p_adj_ ≤ 0.05) with subject study metadata, focusing on metrics most closely related to disease progression status (progressor or non-progressor), including progressor severity (change in EDSS), initial EDSS, final EDSS, diagnosis/MS-subtype, DMT usage at the time of fecal collection, and sex (Fig. S2 and Tables S8–S13). Significantly associated genera and ASVs (as a taxonomic best-hit) are shown in Fig. 3A,B, respectively, and at all other taxonomic ranks in Fig. S2. For each taxon associated with progressor status, abundance (center log ratio (CLR) transformed) (Fig. 3c–j) and prevalence (ratio of subjects positive for a given taxa) (Fig. 3k–r) are shown. Consistent with differential abundance analysis (Fig. 2), a reduction in *Eubacterium* was associated with disease progression (Fig. 3a,b,h,p), while *Alloprevotella* and *Rhodospirillales* were positively correlated with progression (Fig. 3a,b,d,e,l,m). In addition, an expansion of the *Bilophila* was positively associated with both progressor status and severity (genus and/or ASVs) (Fig. 3a,b,f,n). Novel genera correlating with progressor status included a positive correlation with *Sutterella* and negative correlation with *Defluviitaleaceae UCG-011* and *Lactobacillaceae* as a familial best-hit (Fig. 3a–c,g,i,k,o,q). Notably, only minor overlap occurred between correlates of DMT exposure and the genera and ASVs specifically associated with progressor status, suggesting that while therapeutics induce unique shifts in the gut flora, correlations of specific taxa with progressor status are not significantly confounded by treatment status in this patient cohort (Figs. 3a,b, S2, and Tables S12, S13). Interestingly there was also minimal overlap between microbial taxa associated with initial or final EDSS and those associated with progressor status, while *Alloprevotella, Rhodospirillales, Eubacterium ventriosum,* and *Lactobacillaceae* were consistently correlated with progressor severity (change in EDSS) (Figs. 3a,b, S2 and Tables S12, S13). To exclude the effect of potential confounders which could impact the taxa associated with disease progression and/or severity as identified by correlation analysis above, we employed linear mixed model analysis implemented using LinDA^53^. We included initial EDSS, diagnosis, DMT usage, and sex as covariates in the model, followed by identification of taxa associated with progressor status. Notable overlap was observed, with 58% (18/31) of differentially abundant taxa identified by Spearman’s correlation also identified using LinDA, including depletion of *Akkermansia* and elevation of *Rhodospirillales* ASVs (Fig. S9a–d). Taken together these data suggest that microbial correlates of disease progression have a degree of specificity and display minimal overlap with closely related subject metadata, including DMT usage.

To determine if altered microbial abundance of the above identified taxa is sufficient to predict probability of disease progression, median abundance of taxa correlated with progressor status (Fig. 3a,b) was used to stratify patients (high/low abundance) and Kaplan–Meier analysis was performed. Binning patients independent of progressor status by low or high abundance of positively correlated genera, including *Sutterella*, *Alloprevotella*, *Rhodospirillales*, and *Bilophila* was sufficient to significantly stratify patient probability of disease progression over total patient disease duration, suggesting these genera may be useful as a predictive biomarker for future patient disease course (Figs. 3s–v and S3). Conversely, *Eubacterium ventriosum,* which was negatively correlated with progressor status, displayed an inverse relationship in predicting disease progression (Fig. 3w and S3). Taken together, these results reveal specific microbial taxa associated with MS disease progression, whose abundance, when taken individually, has modest potential to predict disease outcome.

## *Vitamin K*_*2*_* and short-chain fatty acid production are depleted in the inferred metagenome of MS progressors*

**Figure 4:**
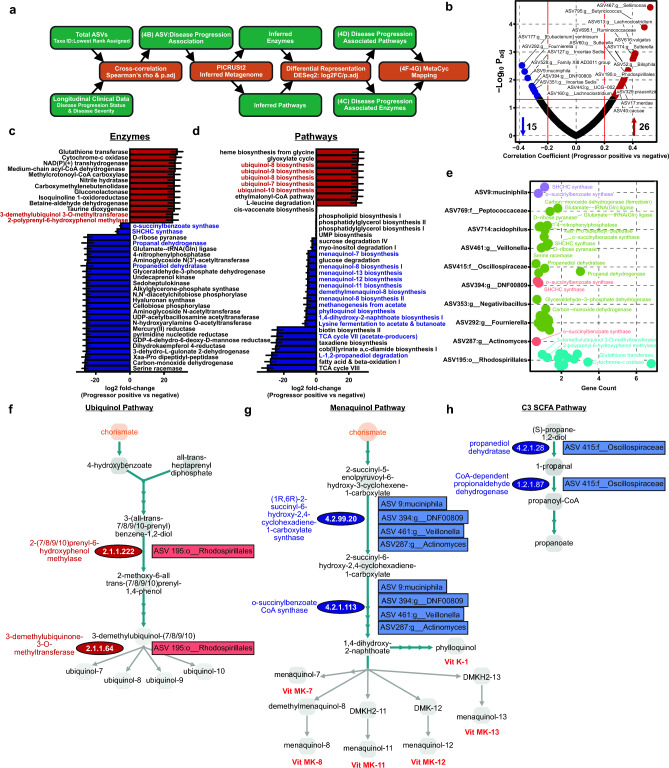
Disease progressors exhibit distinct functional gut microbial metabolic potential. (**a**) Schematic of the computational approach used to infer the functional potential of differentially abundant microbiota associated with disease progression. (**b**) Volcano plot of ASVs (represented as taxonomic best-hits) associated with disease progression as determined by Spearman rank correlation ≥|0.2|, at p_adj_ ≤ 0.05. (**c**) Differentially abundant enzymes and (**d**) pathways in the inferred metagenome of progressor status associated ASVs. Total metagenomic potential was inferred with PICRUSt2 and differential abundance analyzed for the subset of ASVs associated with progressor status using DESeq2 at p_adj_ ≤ 0.05. (**e**) Top 50 differentially abundant enzymes plotted as gene count by ASV (taxonomic best-hit) colored by phylum, as determined by log2 fold-change at p_adj_ ≤ 0.05. Ubiquinol (**f**), menaquinol (**g**), and propanediol degradation (**h**) pathway schematics mapped using MetaCyc and annotated with differentially abundant enzymes and their originating ASVs.

To assess potential functional consequences of the identified changes in microbiota associated with disease progression, total ASVs found to be correlated (Spearman rank correlation cutoff at rho ≥|0.2|, p_adj_ ≤ 0.05) with progressor status were analyzed using PICRUSt2^54,55^ to infer their metagenomic functional potential (enzyme commission numbers (E.C.)/designation, KEGG orthology IDS, pathways), followed by differential abundance testing using DESeq2 (at p_adj_ ≤ 0.05) and ASV:E.C.:pathway mapping using MetaCyc^56^. A detailed description of pathway analysis is presented in the Materials Methods section and supporting data are provided in Supplemental Tables S14–S17, with a graphical schematic of analytical approaches provided in Fig. 4a. In total 41 ASVs were associated with progressor status, with 15 that were negatively correlated (reduced abundance) and 26 that were positively correlated (increased abundance) in the baseline gut microbiome of patients that went on to exhibit disease progression (Fig. 4b and Table S13). Progressor status associated ASVs yielded a predicted metagenome encoding 1239 enzymes (Table S14) encompassing 253 unique pathways (Table S15). Differential abundance testing identified a total of 137 enzymes, 69 of which were predicted to be overrepresented and 68 that were underrepresented (Fig. 4c and Table S16) within the inferred metagenome of progressor microbiota, comprising 35 differentially abundant pathways, 10 enriched and 25 depleted, respectively (Fig. 4d and Table S17). Interestingly, a number of pathways predicted to be enriched in the metagenome of progressors were involved in ubiquinol biosynthesis, typically associated with bacterial aerobic respiration, while several depleted pathways suggested a bias towards anaerobic respiration, namely biosynthesis of menaquinol/phylloquinol, the reduced forms of Vitamin K_2_ (Fig. 4d,e)^57,58^. Further, baseline progressor gut microbiota were depleted in the capacity to synthesize C2, C3, and C4 short-chain fatty acids (SCFA), including a marked reduction in acetate-production, lysine fermentation to acetate and butanoate, and propanediol degradation to propionate (Fig. 4d). To identify putative bacterial species responsible for enriched or depleted enzymes and pathways, the inferred metagenome associated with progressor status was stratified by ASV of origin, and subsetted to include only ASVs and predicted enzymes statistically associated with progressor status (Fig. 4e and Table S18). Segregating ASV by EC gene count revealed a total of ten taxa responsible for the top 50 differentially abundant enzymes (Fig. 4e), including several ASVs previously shown to be enriched (*Rhodospirillales*, Fig. 2f) or depleted (*Akkermansia*, Fig. 2e,f, and *Oscillospiraceae*, Fig. 2f) in the progressor microbiome. To visualize relationships among select prominent pathways, enzymes, and ASVs, differentially abundant pathways and enzymes were mapped with MetaCyC, manually collapsed at shared nodes, and annotated with progressor status associated ASVs (Fig. 4f–h). Interestingly, it was readily apparent that ubiquinol and menaquinol pathways both stem from bacterial chorismate metabolism. Consequently, catabolism of chorismate may represent a critical metabolic branchpoint wherein the over- or under-representation of specific microbiota may favor vitamin K_2_ production (menaquinols) over ubiquinol pathways. Within the ubiquinol superpathways (overrepresented in progressors), a *Rhodospirillales* ASV was predicted to encode two critical enzymes, 2-polyprenyl-6-hydroxyphenol methylase (E.C:2.1.1.222), converting prenyl-benzene-diols into their phenol counterparts, and 3-demethylubiquinol 3-O-methyltransferase (E.C:2.1.1.64), responsible for final ubiquinone production. Interestingly, the same *Rhodospirillales* ASV was also predicted to encode seven copies of glutathione transferase, potentially important for countering bacterial ubiquinone-dependent aerobic metabolism-induced oxidative stress (Fig. 4e,f). Underrepresentation of menaquinol/phylloquinol pathways in progressors was driven by four ASVs, *A. muciniphila*, a *Coriobacteriia* member (genus DNF00909), an ASV from the *Veillonella* genus and an ASV from the *Actinomyces* genus which were predicted to encode 2-succinyl-6-hydroxy-2,4-cyclohexadiene-1-carboxylate synthase (E.C:4.2.99.20) and o-succinylbenzoate synthase (E.C.:4.2.1.113), involved in 2-succinylbenzoate production from chorismate for downstream menaquinol biosynthesis. To identify microbiota with a role in restricting SCFA production in MS progressors, the propanediol degradation pathway was also mapped. *Oscillospiraceae* (known SCFA producers) were predicted to encode essential enzymes in this pathway and, consistently, their abundance was reduced in the baseline microbiome of progressors (Fig. 4b,e,h). Coupled with the numerous *Lachnospiraceae* ASVs depleted in disease progressors (Fig. 2f), as other prominent SCFA producing microbiota, these data are suggestive of diminished SCFA producing potential of the progressor associated microbiota. Together, these data identify candidate species sufficient to induce a bacterial metabolic signature of MS disease progression, which is hallmarked by a reduction in vitamin K_2_ and SCFA production with an increase in aerobic respiration, potentially indicative of elevated oxidative stress within the GI lumen; all of which represent hypotheses to be tested in future studies.

### Baseline gut microbiota are sufficient to predict MS progression

**Figure 5:**
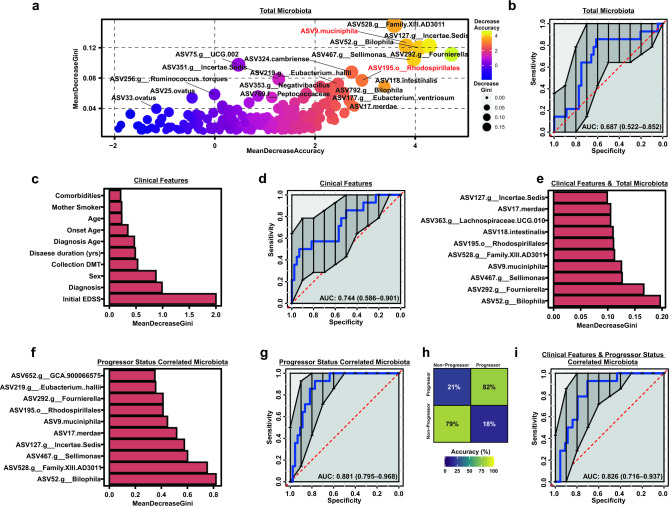
Microbiota composition and patient clinical features are sufficient to predict patient progressor status. (**a**) X–Y scatter plot reflecting total variables of importance by mean decrease in accuracy and mean decrease in Gini coefficient as determined by a Random Forest classifier trained on total ASV level 16S abundance data, with ROC curve analysis shown in (**b**). (**c**) Top 10 clinical features as variables of importance by mean decrease in Gini coefficient as determined by a Random Forest classifier trained on patient metadata available at study baseline, with associated ROC curve shown in (**d**) for clinical data alone and (**e**) when combined with total ASV 16S data. Top 10 variables of importance (**f**), ROC curve (**g**) and class error (**h**) are shown for a Random Forest classifier trained on only the ASVs correlated with progressor status as in Fig. 4B. Inclusion of patient baseline clinical metadata in classifier training of progressor associated microbiota is shown in (**i**). All Random Forest classifiers were bootstrapped 1000 times using a balanced bagging approach with leave-one-out cross validation.

Given that individual ASVs provided modest capacity to predict disease progression (Fig. 3), we next sought to discern the combined predictive capacity of the gut microbiota as a whole. Specifically, to determine if the baseline gut microbiota and/or clinical features were sufficient to predict disease progression, we leveraged Random Forest-based predictive modeling by training classifiers on: (1) ASV-level total microbiota features, (2) clinical features available at baseline (e.g. excluding final EDSS, progressor status, and progressor severity), (3) ASV-level total microbiota combined with baseline clinical features, (4) restricting microbiota to only progressor status correlated ASVs as identified in Fig. 4a and (5) progressor status correlated ASVs combined with clinical features. All classifiers were bootstrapped 1000 times using a balanced bagging approach with leave-one-out cross validation. A detailed description of predictive modeling is described in the materials and methods section. Using the first approach with total microbiota ASVs, *A. muciniphila*, *Rhodospirillales*, *Sellimonas*, and two ASVs belonging to the *Clostridia* class were identified as contributing most significantly to model performance, at a maximum mean decrease in Gini coefficient of 0.15 and mean decrease in accuracy of 3.58 per single ASV (Fig. 5a). Receiver operator characteristic (ROC) curve analysis resulted in a moderate area under the curve (AUC) of 0.687. Interestingly, classifiers built on baseline clinical features alone (approach number two), performed similarly to total microbiota, driven by initial EDSS, diagnosis, and sex (mean decrease in Gini coefficient of 2.0, 0.99 and 0.70 respectively) with an AUC of 0.744 upon ROC analysis (Fig. 5c,d). Combining ASV-level total microbiota and clinical features (approach number three) failed to further enhance the predictive power of the model over that observed using clinical metadata alone, at an AUC 0.695 (Fig. S4), with microbiota features contributing more significantly to overall model performance (Fig. 5e). As a fourth approach, to enhance the predictive capacity of the microbiota, classifiers were also trained on just those ASVs identified as associated with progressor status (Fig. 4b), which improved model performance, resulting in a final AUC of 0.881 in ROC analysis (Fig. 5f,g) and at an out-of-the-box error of 18.9%, with 21% and 18% class errors for progressors and non-progressors, respectively (Fig. 5h). ASVs responsible for the greatest mean decrease in Gini coefficient were consistent with modeling of the total microbiota, including *Akkermansia* and *Rhodospirillales* within the top 10 features of importance (Fig. 5f). Consistent with total microbiome trained classifiers, inclusion of clinical metadata along with progressor correlated ASVs (approach number five) failed to enhance classifier performance (Fig. 5i). Taken together, as proof of principle in a small well-defined cohort, these data suggest that the baseline microbiota and patient clinical features may be sufficient to predict subsequent patient disease progression, with *Rhodospirillales* and *Akkermansia* contributing robustly to model performance.

### Constipation associated gut microbial changes are modest and independent of progressor status

**Figure 6:**
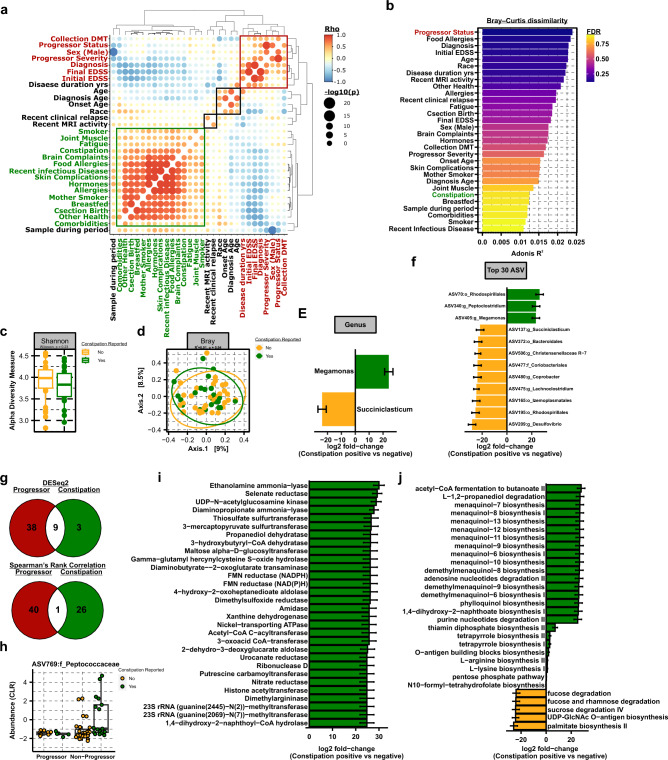
Constipation is associated with a unique microbial signature that is divergent from that of disease progression. (**a**) Metadata bubble plot correlation matrix using a Spearman rank correlation with a significance cutoff at p_adj_ ≤ 0.05, with significance of association denoted by increasing size. (**b**) PERMANOVA of Bray–Curtis dissimilarity with the percent of variance explained by each metadata feature as R^2^ on x-axis and colored by FDR, as determined by the *adonis* test. (**c**) Alpha (Shannon) and (**d**) beta (Bray–Curtis dissimilarity) diversity analysis in subjects not reporting or reporting constipation, as analyzed using Wilcoxon rank sum non-parametric or *adonis* tests, respectively. Differentially abundant taxa by (**e**) genus and (**f**) ASVs represented as taxonomic best-hit between subjects with or without constipation, as determined using DEseq2 using a cutoff of p_adj_ ≤ 0.05. Log2 fold-change reflects increased abundance in subjects experiencing constipation when positive and decreased abundance when negative. (**g**) Venn diagrams of shared or divergent ASVs between disease progressors and subjects experiencing constipation as determined by DESeq2 (p_adj_ ≤ 0.05) and Spearman rank correlation ≥|0.2|, at p_adj_ ≤ 0.05. ASVs associated with both progressor status and constipation are plotted as CLR transformed abundance for shared ASVs in (**h**). (**i**) Differentially abundant enzymes and (**j**) pathways from the inferred metagenome of constipation associated ASVs. Total metagenomic potential was inferred with PICRUSt2 and differential abundance analyzed for the subset of ASVs associated with constipation using DESeq2 at p_adj_ ≤ 0.05.

Detailed metadata on subject demographics, clinical history and any comorbidities was collected from all 58 participants (Tables 1 and 2). To broadly determine the relationships between each feature, metadata was cross correlated (Spearman rank correlation, cutoff at rho ≥|0.2|, p_adj_ ≤ 0.05) for hierarchical clustering (Ward.D2 squared minimum variance method) (Fig. 6a). Four main feature clusters were observed in metadata correlation, including a small cluster consisting of an intuitive relationship between recent clinical relapse and recent MRI activity (black border), a second cluster defining relationships between disease progression, disability status, sex, and DMT usage (red border), a third cluster segregating disease duration, age of onset/diagnosis and race (black border), and finally a large cluster consisting primarily of patient reported symptoms, including brain complaints, fatigue, and joint or muscle pain (green border). Interestingly, this final cluster also included facets of childhood history (c-section births, breastfeeding and maternal history) as well as allergies (general allergies, food and skin complications). Given that some patient reported metadata features correlated with progressor status, the effect on microbiome structure, as measured by beta diversity, was assessed for each feature as potential confounders. Specifically, the PERMANOVA effect size (as determined by *adonis* R^2^ value) of metadata features associated with Bray–Curtis dissimilarity of beta diversity (p_adj_ ≤ 0.05) was assessed and plotted as a bar graph (Figs. 6b and S5). While none of the metadata features passed FDR constraints, progressor status trended towards significance with a modest impact on overall microbial diversity (R^2^ = 0.02, FDR = 0.06), indicating that covariates of progressor status and other patient characteristics do not represent major confounders in this patient cohort.

**Table 2:** Self-reported symptoms and additional patient history.

Reported n, (%)	Non-progressor n = 45	Progressor n = 15	Total n = 60	Statistical analysis
Constipation reported	8.0 (18.2%)	2.0 (14.3%)	10 (17.2%)	p = 0.11
Brain complaints	18.0 (40.9%)	7.0 (50.0%)	25.0 (43.1%)	p = 0.04
Fatigue	8.0 (18.2%)	6.0 (42.9%)	14.0 (24.1%)	p = 0.61
Vitamins or dietary supplements	14.0 (31.8%)	7.0 (50.0%)	21.0 (36.2%)	p = 0.19
Antibiotic use in the past year	10.0 (22.7%)	3.0 (21.4%)	13.0 (22.4%)	p = 0.09
Joint or muscle pain	11.0 (25.0%)	7.0 (50.0%)	18.0 (31.0%)	p = 0.48
Recent infectious disease	12.0 (27.3%)	5.0 (35.7%)	17.0 (29.3%)	p = 0.14
Other reported health conditions	11.0 (25.0%)	2.0 (14.3%)	13.0 (22.4%)	p ≤ 0.01
Sample collected during menstrual cycle	1.0 (2.3%)	2.0 (14.3%)	3.0 (5.2%)	p > 0.9999
Hormone use in the past yr	8.0 (18.2%)	2.0 (14.3%)	10.0 (17.2%)	p = 0.11
Current smoker	9.0 (20.5%)	3.0 (21.4%)	12.0 (20.7%)	p = 0.15
Biological mother smoked	13.0 (29.5%)	5.0 (35.7%)	18.0 (31.0%)	p = 0.10
Born via c-section	4.0 (9.1%)	1.0 (7.1%)	5.0 (8.6%)	p = 0.38
Breastfed by mother	15.0 (34.1%)	6.0 (42.9%)	21.0 (36.2%)	p ≤ 0.05
Food allergies or sensitivities	7.0 (15.9%)	3.0 (21.4%)	10.0 (17.2%)	p = 0.34
Skin complications	11.0 (25.0%)	4.0 (28.6%)	15.0 (25.9%)	p = 0.12
Seasonal or environmental allergies	19.0 (43.2%)	4.0 (28.6%)	23.0 (39.7%)	p ≤ 0.01
Other comorbidity reported	9.0 (20.5%)	0.0 (0.0%)	9.0 (15.5%)	p ≤ 0.01

Gastrointestinal (GI) symptoms are frequently suggested to be confounding determinants of MS-specific gut microbial signatures^34,35^. Consequently, we analyzed the effect of patient reported constipation, as the most frequent GI complaint among MS patients^36^, on the gut microbiome, for comparison to alterations associated with progressor status. Consistent with progressor status diversity analysis (Fig. 1l,m) and permutational analysis of Bray–Curtis dissimilarity (Fig. 6b), no overt difference in alpha or beta diversity were driven by constipation (Fig. 6c,d). Direct fold-change analysis of genera and ASV taxonomic best-hits identified a limited number of taxa that were differentially abundant in patients with constipation, which did not significantly overlap with microbiota that were altered in disease progression (Fig. 6e–g). Specifically, only two genera, one increased and one decreased, were associated with constipation, while a total of twelve differentially abundant genera (two increased and ten decreased) were observed with progressor status, with only *Succinicalsticum* conserved between the two states (Figs. 6e and 2e). Interestingly, given their known role in propionate production, diminished abundance of *Succinicalsticum* may suggest that constipation, as a major symptom of MS, may further drive a reduction in beneficial SCFA-producing microbiota. The abundance of only a total of twelve ASVs (taxonomic best-hit) were altered by constipation (Fig. 6f), nine of which were shared in common with shifts occurring with progressor status (Fig. 6g). We previously used Spearman rank correlation ≥|0.2|, p_adj_ ≤ 0.05 to identify individual ASVs associated with progressor status, which we applied here to identify ASVs associated with constipation. Comparison of ASV taxonomic best-hits revealed only a single shared ASVs associated with both constipation and progressor status (Fig. 6g,h and Table S13), ASV769 in the family *Peptococcaceae*. Direct comparison of the abundance profile of ASV769 revealed a modest decrease associated with constipation, which occurred more substantially with disease progression (Fig. 6h). To determine if microbial functional profiles rather than individual taxa or ASVs were similar between gastrointestinal symptoms and disease progression, the metagenome of ASVs associated with constipation was inferred using PICRUSt2 for comparison to progressor pathway analysis (Fig. 4). In total, 304 enzymes were determined to be differentially represented in the metagenome of subjects experiencing constipation (Table S19). Of these 304 enzymes, 16 were shared with enzymes predicted to be over or underrepresented in disease progressors, but only three of these enzymes displayed concordant directionality (increased in both constipation and disease progression including EC:2.7.7.65: Diguanylate cyclase, EC:6.5.1.6: DNA ligase and EC:6.5.1.7: DNA ligase) (Figs. 4c,e, 6i). Interestingly, all three enzymes were driven by higher abundance of ASV106:g_*Faecalitalea* from the *Erysipelotrichaceae* family in constipation, while in disease progression, ASV195:o__*Rhodospirillales* expansion was responsible (Fig. 4e and Table S18). Similar to comparison of differentially represented enzymes, there was minimal overlap in pathway analysis between subjects experiencing constipation and disease progression (Fig. 4d, 6j, and Table S20). A total of 56 pathways were altered in the metagenome of patients reporting constipation, with 13 pathways shared with progressors and only a single pathways displaying consistent direction of change (sucrose degradation IV, decreased in both) (Table S20). Interestingly, menaquinol pathways were increased as associated with constipation (opposing the notable decrease observed in disease progression), owing to expansion of two *Coriobacteriia* family members, ASV371:f__*Eggerthellaceae* and ASV654:g__*Parvibacter*, suggesting independent mechanisms leading to menaquinol dysregulation in constipation and disease progression. Taken together, these data suggest that constipation is not a major confounder of progressor status associated shifts in the gut microbiome or their inferred functional potential. Further, while constipation is a common clinically reported symptom in MS, in this cohort, the impact to overt community richness and composition of the gut microbiome was modest as compared to MS disease progression, with each displaying a unique microbial and metagenomic signature.

## Discussion

**Figure 7:**
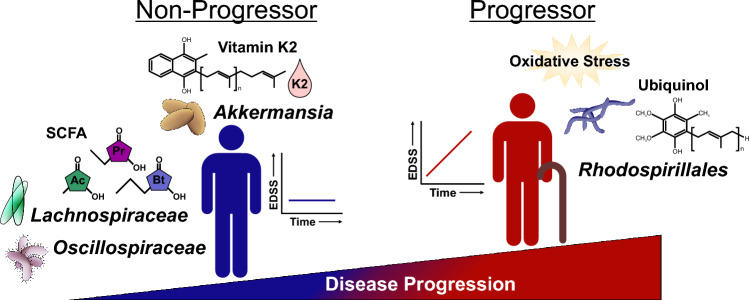
Summary of gut microbiota related mechanisms associated with MS disease progression. Our data and analysis revealed a significant enrichment in oxidative stress-inducing aerobic respiration at the expense of microbial vitamin K2 production (linked to *Akkermansia*), and a depletion in SCFA metabolism (linked to *Lachnospiraceae* and *Oscillospiraceae*) associated with MS progression.

We leveraged longitudinal tracking of disease severity EDSS changes, coupled with baseline assessment of the fecal gut microbiome, to identify candidate microbiota predictive of risk of disease progression. Pathway analysis on the inferred metagenome of taxa associated with progression revealed a significant enrichment in oxidative stress-inducing aerobic respiration at the expense of microbial vitamin K2 production, and a decrease in SCFA metabolism (Fig. 7). Our study not only showed gut microbial signatures could be used as potential predictors for disease progression, but also linked functional pathways unique to progression. Importantly, only a single previous study leveraged change in EDSS to measure disease progression for correlation with gut microbiome composition, although a metabolic network analysis was not performed^51^. Specifically, Devolder et al. assessed change in EDSS over a 4.4-year period, collecting baseline fecal samples from MS patients. Consistent with our findings, metrics of microbial diversity and community structure were not different between patients who experienced disease progression and those that did not^51^. Additionally, disease worsening was associated with an enterotype replete with *Bacteroides*^51^, echoing increased abundance of Bacteroidota phylum members *Alloprevotella* and *Prevotella_9* genera (Fig. 2e) in our patient cohort, as well as consistent depletion of *Desulfovibrio* and *Oscillospiraceae* associated with disease worsening (Fig. 2f)^51^.

The majority of other studies characterizing disease activity-associated microbiota, either compared gut microbiome composition in clinical relapse, between RRMS and progressive forms of disease, or associated microbiota with EDSS at a given cross-sectional timepoint in patient disease course. Thirion et al. reported an elevation in several *Clostridiales* members and a depletion of *Actinomycetales* member *Gordonibacter urolithinfaciens* species linked to active disease with relapse (Fig. 2c). Interestingly, a decrease in *Faecalibacterium prausnitzii* (*F. prausnitzii*) was also associated with increased disease activity^38^, consistent with our own results wherein *F. prausnitzii* positively associated with disease progression (Fig. 3b and Table S13). Interestingly, *F. prausnitzii* was also identified as depleted in MS patients in the largest case–control MS microbiome study to date^37^. Cox et al. identified an elevation of *Clostridiales* members and depletion of *Ruminococcus*, *Oscillibacter*, and *Lachnospiraceae* species associated with worsening disease in RRMS or PMS, consistent with our own analyses of the baseline progressor microbiome (Figs. 2, 3)^59^. Interestingly, *A. muciniphila*, as the single member of the Verrucomicrobia phylum, was also reported to be depleted in progressive disease, a finding that was also observed in the recent International MS Microbiome Study (iMSMS) and in our own study herein^37,59^. Given that *Akkermansia* represents the single bacterial species that is perhaps most consistently reported to be increased in MS patients as compared to healthy controls^19,20,37,60–64^, this was an unexpected finding. However, increasing evidence suggests that rather than serving as a disease driver, enriched abundance of this species perhaps serves as a compensatory and/or protective mechanism specific to MS^65^. Our own data identifying a correlation between reduced abundance of *Akkermansia* and progressor status (Fig. 3b) indeed fits with this model. An alternative explanation may be that *Akkermansia* is increased (in MS relative to the healthy control populations) early during the MS initiation phase, with decreasing abundance upon subsequently worsening disease. How the fluctuation of *Akkermansia* abundance is regulated by the host and any interplay of these mechanisms with MS has yet to be established. However, it is possible that *A. muciniphila* is associated with modulation of mucus production, functioning to restore gut barrier integrity and appropriate tight junction assembly to prevent dysbiosis^66,67^. Therefore, a reduction is seen in progressors.

Studies have identified a change in *Prevotella* abundance in the MS gut microbiome, including eight studies reporting a decrease in abundance^19,20,28,41,63,68–70^. Consequently, *Prevotella* was tested in preclinical models as a potential probiotic^71,72^. In contrast, the iMSMS cohort study reported a positive association between several *Prevotella* species and severity of disease in PMS^37^, echoing our own data wherein the *Prevotella-9* genus is expanded in disease progressors. Collectively, these data are reminiscent of the above described relationship between *Akkermansia* abundance and disease progression, underscoring the notion that signatures of the gut microbiome in MS may differ from those alterations associated with (or contributing to) disease progression.

No studies have previously documented an increase in the *Rhodospirillales* order in MS. However, recent findings from the International MS Microbiome Study (iMSMS) revealed a correlation between the *Rhodospirillum* genus and cross-sectional disease severity, albeit in opposing directions from our findings, with increased abundance in patients with RRMS and decreased abundance in PMS^37^. In an independent cohort, *Alphaproteobacteria* (class), of which *Rhodospirillium* is a member, was also found to be increased in RRMS^19^. Furthermore, *Rhodospirillaceae* (family) has been linked to Alzheimer’s disease and with a higher risk of irritable bowel syndrome, suggesting a broader association with neurological disease and inflammation^73,74^. *Rhodospirillales* have also been positively associated with segmented filamentous bacteria, known inflammatory Th17 inducers in the gut, with a posited role in MS pathogenesis^75^. Interestingly, antibiotic treatment in an experimental autoimmune uveitis model resulted in a reduction of *Alphaproteobacteria*, correlating with increased *Gammaproteobacteria*, collectively diminishing the Th17 response and enhancing regulatory T cell proliferation^76^. These findings parallel our own study, wherein *Rhodospirillales* (*Alphaproteobacteria* class) is contracted in non-progressors with a corresponding expansion of *Gammaproteobacteria* (Fig. 2).

Though one prior study examined gut microbiome composition in relationship to EDSS changes^51^, functional mechanistic understanding of microbiome changes was lacking. We conducted inferred metagenomic analysis and revealed an imbalance between bacterial ubiquinol and menaquinol biosynthetic pathways (Fig. 4C–G). Interestingly, increased ubiquinol at the expense of bacterial menaquinol, as we observed in MS progressors, is a known signature of enhanced bacterial reliance on aerobic respiration in the inflamed gut where oxygen levels are increased^57,58,77–82^. Moreover, facultative anaerobes, including *Rhodospirillales* and other Proteobacteria phylum members, expand under conditions of inflammation and increased luminal oxygen, where they garner a fitness advantage^31,83–89^. Consistently, *Rhodospirillales*-driven enrichment of 2-(7/8/9/10)prenyl-6-hydroxyphenol methylase (E.C. 2.1.1.222), involved in ubiquinol biosynthesis, was increased in the baseline gut microbiome of patients that would later exhibit disease progression and the single responsible ASV was also predicted to encode glutathione transferase (E.C. 2.5.1.18), necessary to combat oxidative stress induced during aerobic respiration (Fig. 4e,f). These data suggest that an early predictor of subsequent MS progression may be inflammation in the gut, triggering increased luminal oxygen availability that favors outgrowth of facultative anaerobes, such as *Rhodospirillales* and other *Proteobacteria*, thus driving overall gut microbiome imbalance.

Increased oxygen-dependent aerobic respiration in the gut has also been linked to a depletion of SCFA-producing obligate anaerobes, which lack the capacity to withstand elevated luminal oxygen^30,31^. Consistently, we observed a depletion of pathways involved in acetate, propionate, and butyrate production associated with MS progressors and diminished abundance of SCFA-producing microbiota, including *Lactobacillaceae*, *Oscillospiraceae*, and *Lachnospiraceae* members (Figs. 2e,f, 3a,b and 4d). To highlight the importance of this finding, we identified individual ASVs, depleted in the MS-progressor gut microbiome, that could be causally linked to a reduction in essential genes responsible for propanoate production (Fig. 4h). Notably, fecal metabolomics and metagenomics analyses previously have identified markers of elevated oxidative stress and a reduction in SCFA abundance associated with RRMS and SPMS^90^. Moreover, reduced fecal SCFA have been consistently reported in other studies including a suggestion of their restoration upon DMT usage^37,41^. However, dysanaerobiosis, characterized by the increase in oxygen availability within the lumen of the gastrointestinal tract, as a main driver of SCFA-producer reduction, represents a novel emerging mechanism for future study of the MS microbiome, particularly as relevant to disease progression.

In addition to its role in anaerobic respiration for the microbiota, menaquinol biosynthetic pathways are also involved in the production of vitamin K^91^. Interestingly, Lasemi et al. reported strikingly lower levels of serum vitamin K2 (VK2) in MS patients (235 ± 100 ng/ml vs. 812 ± 154 ng/ml in healthy controls) and that these levels were further reduced in women. Moreover, waning VK2 was linked to relapse and lesions in the CNS^91^. Functionally, vitamin K improves mitochondrial dysfunction^92^, inhibits the production of reactive oxygen species to protect neurons and oligodendrocyte precursors from injury^93,94^, and is present at higher concentrations in myelinated regions of the brain, with a role in sphingolipid metabolism^95–98^. Further, there is evidence that vitamin K is beneficial in other neurological diseases, including Parkinson’s^99^ and Alzheimer’s disease^100^. In experimental models of MS, vitamin K increases CNS sulfatides, an essential lipid component of myelin^101^, and reduces disease severity when administered prophylactically^102^. Importantly, although a well-known major contributor to bioavailable vitamin K is the gut microbiota, a species-specific link to depletion in MS has not been established. Here, we identified two essential genes involved in vitamin K production contributing to a depletion in menaquinol biosynthesis (Fig. 4c–e,g) that were differentially abundant in the inferred metagenome of patients experiencing disease progression. Moreover, we linked these genes to a reduction of four bacterial taxa depleted in the MS-progressor gut microbiome, including, most notably, *Akkermansia*. Consequently, we propose a novel connection between altered *Akkermansia* abundance (one of the most consistent hallmarks of the MS gut) and a reduction in vitamin K, as an important area for future research.

To determine if constipation was a major confounder in our own study cohort, we compared the gut microbiome of patients reporting constipation to those exhibiting progression of disease. We observed minimal overlap between taxa associated with progression compared to taxa associated with constipation, including their inferred functional potential. These data suggest that GI dysfunction is not a major contributor to MS progression driven shifts in the microbiota within our study cohort, which is also consistent with approximately equal incidence of constipation in progressors vs. non-progressors (Table 2). Interestingly, there are some overlaps between the microbiota associated with constipation here and gut microbiome signatures of MS identified in other patient cohort case-control studies. For example, an increase in *Blautia* has been observed in four previous studies^19,39,42,69^. Our data suggest that decreased *Blautia* may indicate gastrointestinal dysfunction in MS patients (Table S13), which is supported by previous work establishing that *Blautia* is also increased with age, BMI, and GI symptoms in MS patients specifically^103,104^. Similarly, two previous studies^37,70^ reported a decrease in *Bacteroides thetaiotaomicron*, which has also been linked to diet, digestion, and constipation, specifically through stimulation of gut motility^70,105,106^. This aligns with our own analysis, which identified a decrease in *B. thetaiotaomicron* in patients experiencing constipation. Interestingly, several genera exhibited an inverse relationship with known alterations in the MS gut microbiome as compared to constipation-associated shifts highlighted here. For example, while *Megamonas* is depleted and^28,90^ *Alistipes* species^59,90^, is enriched in MS, we observed the opposite with constipation. Lastly, *Odoribacter splanchnicus* and *Erysipelotrichaceae*, such as *Faecalitalea,* have been previously associated with GI complaints (in a non-MS cohorts), underscoring the specificity in gut microbiome signatures specific to constipation^107,108^. Taken together, our results identify a distinct microbial signature of constipation in MS patients, which, as highlighted here, can be applied to future and past cross-sectional case–control studies to de-confound comparisons between healthy individuals and people with MS, who frequently report constipation.

The use of microbiota abundance as a predictive non-invasive and inexpensive biomarker of disease progression is an attractive concept. To determine any predictive value of the microbiota in MS progression, we took two approaches: (1) leveraging association with disease progression to identify candidate microbiota for subsequent risk assessment using stratification by abundance of a single taxon, and (2) machine learning to identify baseline microbiota signatures of most utility in segregating patients who progress from those who do not. In the first approach, *Sutterella*, *Alloprevotella*, *Rhodospirillales*, *Bilophila* and *Defluvittaleaceae* abundance was sufficient to stratify patient risk of disease progression (Fig. 3). Interestingly, increased *Sutterella* abundance associated with risk of disease progression has been reported previously^70^. However, *Sutterella* levels are also contracted in MS patients as compared to healthy controls^28^, and increased abundance has been associated with immunomodulatory treatment intervention, postulated to normalize microbiome composition^20^. Moreover, *Sutterella* is associated with the HLA-DQ8 class II haplotype, which is present at increased frequency in MS and specifically suggested to play a role in disease progression^109–112^. Interestingly, high *Alloprevotella* abundance, associated with disease progression in our study, has also been associated with the DQ8 haplotype^113^, though *Alloprevotella* is far less studied in MS specifically. In machine learning approaches, both *Rhodospirillales* and *Akkermansia* were top contributors to predictive models segregating MS-progressors from non-progressors. Predictive modeling restricted to gut microbial composition performed modestly but comparably to the use of readily available patient clinical metadata, including initial EDSS, patient diagnosis, disease duration, and age of onset. Importantly, combining microbiota and clinical features significantly improved predictive performance of disease progression, as did subsetting microbiota features to include only those identified as associated with disease progression (Fig. 5). These data serve as proof of principle to demonstrate that analysis gut microbiome composition can be predictive of MS-progression, which should be validated by expanding such approaches to additional longitudinal study cohorts.

MS is a highly heterogeneous disease with various clinical subtypes and accompanying disease courses. Longitudinal tracking of disease progression coupled with baseline analysis of the gut microbiome and patient clinical features offers several distinct advantages including: (1) characterization of disease-relevant microbiome changes, (2) identification of microbiome prognostic makers, (3) offering a built-in internal control, (4) identification of pathological mechanisms, and (5) enhanced translational promise. There is an unmet need to identify patients who are at high risk for progression and need more aggressive therapeutic intervention to prevent long-term disability. Notably, early aggressive treatments potentially pose a greater risk for infection and for other complications compared to traditional therapies; therefore, it is of utmost importance to identify the right patient for aggressive therapy to prevent disability. Our study provides evidence of potentially using the gut microbiome as a prognostic indicator, facilitating a more personalized therapeutic approach. Most importantly, our data sheds light on the pathological mechanisms unique to progression, including oxidative stress, Vit K2, and SCFA, which could contribute to worsening disease.

## Study limitations and additional analyses

While the use of 16S RNA gene sequencing offers reliable taxonomic assignment and abundance profiling, shotgun metagenomic sequencing has gained increasing use. In an effort to reconcile these approaches, the recent *Greengenes2* database was specifically developed to unify genomic and 16S rRNA taxonomic assignment, to minimize the disparity between sequencing strategies^114,115^. To determine the extent to which our analyses and interpretation are recapitulated, we performed a re-analysis applying the updated *Greengenes2* database (Fig. S6). Importantly, there was high concordance between original *SILVA* assigned taxonomy and updated *Greengenes2* datasets, and where taxonomy differed, the origin of such deviations was apparent. For example, Firmicutes has been subdivided into Firmicutes A-H, resulting in a loss of significant fold-change within this phylum using Greengenes2. Further, the *Rhodospirillales* ASVs enriched with disease progression consistently classify as *Alphaproteobacteria* between both databases but resolved to the genus level using Greengenes2 (CAJLXD01 and CAG-239). While we recognize that inference of the metabolic potential of the microbiota associated with disease progression could likely be enhanced through the use of shotgun metagenomic strategies, such approaches are still inherently reliant on curation of the same databases to inform functional potential and cannot always be reliably associated with specific bacterial taxa. Consequently, as with any sequencing based strategy, we recognize the need for formal functional validation of all inferred metagenomic analyses presented here, viewing such inferences as fodder for hypothesis driven future mechanistic study.

DMT usage is a frequent confounder of microbiome studies given the known impact of therapeutics on gut microbiome composition^37^. All broad diversity analyses were corrected for DMT usage (Figs. 1m and 6b), moreover, correlation at each taxonomic rank displayed minimal overlap between microbiota associating with both disease progression and DMT usage (Figs. 2 and S2). To further explore the extent to which DMT usage confounded gut microbial signatures of disease progression, we binned study participants, independent of progression and/or MS-subtype, based on DMT usage at the time of fecal collection (Fig. S7). No overt differences in species richness and overall community structure were observed (Fig. S7a,b), suggesting a modest impact of DMT usage on the gut microbiota, as previously determined (Fig. 6B). Direct fold-change and/or correlation comparative analyses between disease progression and DMT usage revealed shared genera (*Alloprevotella*, *Eubacterium* and *Succiniclasticum*) and a total of 18 ASVs (out of a total of 56) in common between the two analytical strategies, all of which displayed a consistent direction of change (Fig. S7c–j). In contrast, inferred metagenomic functional potential of the differentially abundant ASVs associated with DMT usage displayed minimal overlap with disease progression status, with only seven shared enzymes and no consistent pathway enrichment (Fig. S7k,l). Taken together these data suggest that a modest degree of overlap between disease progression status and DMT usage associated microbiota was present within this cohort, and that the functional consequences may be divergent.

Disease progression can be mediated by both inflammatory and non-inflammatory processes which may have distinct gut microbial underpinnings segregating by disease subtype. Such alteration may not have been apparent in the analytical approach taken here- namely binning participants independent of disease subtype based on progression alone. To address this limitation, study participants were instead binned into three groups, BMS (control), RRMS (likely inflammatory), PPMS/SPMS (likely non-inflammatory) for comparison of gut microbial signatures of disease subtype to those observed with disease progression. No differences in species richness or community composition were observed based on disease subtype (Fig. S8a,b) consistent with analysis of disease progression alone. Interestingly, direct fold-change analysis of genera between BMS and either RRMS or PMS revealed a consistent enrichment in *Prevotella_9* abundance as observed with disease progression status as well as disease subtype specific alterations (Fig. S8c,d). Fold-change of individual ASVs suggested that disease subtype captured a portion of the gut microbial alterations observed with disease progression with 13 and 14 shared ASVs in RRMS and PMS respectively. Spearman rank correlation of disease subtype revealed minimal overlaps with progression status, with only 3 ASVs shared in common (ASV415:f__*Oscillospiraceae*, ASV40:*caccae*, ASV177:g*__[Eubacterium] ventriosum group*) all of which shared the same directionality (Fig. S8g,h). Inferred metagenomic analysis of the disease subtype associated ASVs, suggested a consistent enrichment in anaerobic ubiquinol pathways as well as novel CMP-legionaminate biosynthesis, involved in sialic acid host immune evasion and invasion^116^, as the top differentially abundant pathway in both RRMS and PMS (Fig. S8k,l). Taken together these data suggest that gut microbial hallmarks of future disease progression share some, but not all, signatures of MS subtype.

Longitudinal tracking of disability status coupled with the analysis of the baseline gut microbiota represents an advancement in understanding the drivers of disease progression in MS. We aimed to present a proof of principle study of a small well-defined cohort. However, we recognize that incorporating healthy control participants may augment understanding of the gut microbial association with disease progression. Further, future efforts should be aimed at expanding study participant number, coupled with collection of the fecal microbiota early in disease course (ideally from initial diagnosis or in the prodromal phase in high risk or suspected MS cases). Further, incorporation of shotgun metagenomics and fecal and/or serum metabolomics would enhance our understanding of the functional characteristics of the microbiota associated with disease progression. Moreover, longitudinal collection of fecal samples over time, with paired assessment of disability status, coupled with incorporation of a matched male:female ratio, greater study participant diversity and geographical regions, and dietary information would further validate and expand our understanding of gut microbiota dynamics as it pertains to CNS autoimmunity and MS progression specifically.

## Methods

### Recruitment and study design

A total of 58 MS patients of all types (RRMS, BMS, SPMS, PPMS) were recruited from the University of Michigan (UM) Multiple Sclerosis and the Autoimmunity Center of Excellence (ACE). Patients with BMS were defined as having an EDSS score of ≤ 3 at least 15 years after disease onset, with no prior MS treatment and were enrolled as a non-progressor control group. As previously described, only true BMS patients who do not have either physical or cognitive impairments were included^14^. RRMS, SPMS and PPMS were defined by the 2010 Revised McDonald criteria^117^. Participant disability status was evaluated at baseline and 4.2 ± 0.98 years later using the expanded disability status scale (EDSS)^118^. Progressors were defined as EDSS progression of >|0.5| modified from that used in the EXPAND trial^119^. All participants had no evidence of relapse or corticosteroid treatment within 3 months prior to baseline and final EDSS assessment. All participants completed a baseline clinical survey reporting subject demographics, disease subtype and history, medications, and any comorbidities as outlined in Table 1. No patients reported any dietary restrictions or modifications. Informed consent, approved by the University of Michigan Institutional Review Board, was obtained from patients before study participation. All patients were given written informed consent. This study adhered to the tenets of the Declaration of Helsinki and all research was performed in accordance with relevant guidelines/regulations.

### Specimen collection

Stool was self-collected by subjects using the OMNIgene•GUT (OMR-200) kit (https://www.dnagenotek.com/US/products/collection-microbiome/omnigene-gut/OMR-200.html) and then shipped to UM ACE principal investigator lab within 2 weeks. Stool samples were stored at ambient temperature for less than 4 weeks before DNA extraction.

### Stool sample preparation and 16S DNA sequencing

DNA extraction was performed using the Eppedorf EpMotion liquid handling system following the Qiagen MagAttract PowerMicrobiome kit (Qiagen, catalog no. 27500–4-EP). Sequencing libraries were prepared by the University of Michigan Host Microbiome Core as described previously^120^. Following extraction, samples were quantify using the Quant-iT PicoGreen dsDNA Assay kit (Thermo Fisher, catalog no. P7589). The V4 region of the 16 s rRNA gene was amplified from each sample using a dual indexing sequencing strategy in a 20-μl PCR reaction containing 2 μl of 10X Accuprime PCR II buffer (Life Technologies, catalog no. 12346–094), 5 μl of 4 μM universal primers 515-F (5’-GTGCCAGCMGCCGCGGTAA-3’) and reverse 806-R (5’-GGACTACHVGGGTWTCTAAT-3″, 0.15 μl of Accuprime High-Fidelity Polymerase, and 1 μl of DNA template and 11.85 μl of sterile PCR-grade water. PCR cycling conditions were as follows: 2 min at 95 °C, 30 cycles at 95 °C for 20 s, 55 °C for 15 s, and 72 °C for 5 min, with a final extension at 72 °C for 10 min^121^. PCR products were visualized using an E-Gel 96 with SYBR Safe DNA Gel Stain, 2% (Life technologies, catalog no. G7208-02). Libraries were normalized using SequalPrep Normalization Plate Kit (Life technologies, catalog no. A10510-01), the concentration of the pooled samples determined using Kapa Biosystems Library Quantification kit for Illumina platforms (KapaBiosystems, catalog no. KK4824) and the sizes of the amplicons were determined using the Agilent Bioanalyzer High Sensitivity DNA analysis kit (Agilent, catalog no. 5067-4626). The final library consisted of equal molar amounts, normalized to the pooled plate at the lowest concentration and were prepared according to Illumina’s protocol for preparing libraries for sequencing on the MiSeq (Illumina, catalog no. 15039740 Rev. D) for 2 nM libraries. Sequencing was done on the Illumina MiSeq platform, using a MiSeq Reagent Kit V2 500 cycles (Illumina, catalog no. MS102-2003), according to the manufacturer’s instructions with minor modifications^121^ including the use of Accuprime High Fidelity Taq (Life Technologies, catalog no. 12346094) in a 5.5 pM reaction to which a 15% PhiX spike was added. Sequencing reagents were prepared according to the Schloss SOP. Custom read 1, read 2 and index primers are added to the reagent cartridge and FASTQ files were generated for paired end reads^121^.

### 16S data preprocessing and taxonomic assignment

Raw sequencing reads were analyzed using *DADA2* version 1.24.0 in R version 4.2.1^46,122^. Following quality assessment, reads were filtered and trimmed (forward at 240 nt and reverse at 160 nt) and PhiX reads were removed with a maximum number of errors (maxEE) of 2 and quality score of 11. Following standard error learning, forward and reverse reads were merged, and chimeric reads removed using the consensus method. An average of 76.53% total reads were retained at a mean of 168,020 reads per sample. Taxonomy was assigned against the SILVA database (version 138.1)^49^. Unique sequences were aligned using *DECIPHER* version 2.26.0, pairwise distances computed for phylogenetic tree construction with neighbor-joining to fit a maximum likelihood tree using *phangorn* version 2.11.1. The final tree was optimized using a general time-reversible (GTR) model with stochastic rearrangement and rooted using midpoint rooting^123–125^. Taxa, ASV counts, and the final rooted tree were imported into *phyloseq* version 1.40.0 for downstream analysis^50^. Reads that failed to assign at the level of phylum, non-bacterial reads and reads falling below a mean prevalence of 2 where removed, including *Patescibacteria*, *Planctomycetota*, *Spirochaetota*, and *Thermoplasmatota*.

### Diversity analysis

Alpha diversity (Observed, ACE, se.ACE, Shannon, Simpson, InvSimpson, Fisher)^126–130^ was calculated using the estimate_richness function in *phyloseq* version 1.40.0 with Wilcoxon rank sum non-parametric testing between groups. Beta diversity (Bray–Curtis, Unifrac, and weighted Unifrac)^131,132^ was calculated using the distance function in *phyloseq* version 1.40.0 and represented as a PCoA of ASV level data with multivariate analysis of variance (adonis2) using the *vegan* package version 2.6–4^133^.

### Taxonomic summaries and differential abundance testing

Closely-related taxa were agglomerated using single-linkage clustering with the tip_glom() function in *phyloseq*. The AGNES algorithm was used with a numeric scalar of 0.2 as the distance threshold for merging taxa^134^. Taxa were also filtered at a 5% prevalence threshold with a mean abundance threshold of 10 reads per sample. Bar graphs represent the top N taxa at each rank as relative abundance of that taxon. The same filtering parameters were applied for differential abundance testing at each taxonomic rank where taxa were agglomerated using the tax_glom function in phyloseq prior to statistical testing using *DESeq2* with cutoff at p_adj_ ≤ 0.05^52,135^. Prior to fold-change analysis of individual ASVs, a taxonomic ‘best hit’ was assigned using the format_to_besthit function in the *microbiomeutilities* package version 1.00.16^136^. Heatmaps and bar graphs of the abundance and prevalence of taxa were generated using centered log ratio (CLR) transformed data with the comp_heatmap function in *microViz* version 0.10.6^137^. To generate the taxonomic association tree and key, data was normalized using total sum scaling and linear regression was used to model association with progressor status^137,138^. Model statistics including regression coefficient estimate, test statistics and p-values are contained in Table S7. The taxonomic association tree was generated using the taxatree_plots function in *microViz* version 0.10.6^137^. Mixed linear models to adjust for confounders including initial EDSS, diagnosis, DMT usage, and sex were conducted using LinDA for both disease progressor status and disease progression severity using standard settings^53^.

### Correlation of taxa and clinical features

Following agglomerative clustering consistent with differential abundance testing as outlined above, taxa were filtered at a 3% prevalence threshold with a mean abundance threshold of 10 reads per sample, transformed compositionally (CLR transform), and aggregated at each taxonomic rank. Spearman’s rank correlation of clinical metadata and microbiota was performed using the associate function in the *microbiome* package version 1.18.0^139^ and filtered at rho ≥|0.2|, at p_adj_ ≤ 0.05. Abundance and prevalence of correlated phyla and genera are represented as center log ratio transformed and proportional data, respectively. Linear regression modeling of progressor correlated taxa was performed using the *ggpmisc* package version 0.5.2^140^. Spearman’s rank correlation of clinical features alone used the *stats* package version 4.2.1^122^ and was plotted using *heatmaply* version 1.4.2 with a significant cut-off of p_adj_ ≤ 0.05 and Ward.D2 clustering^141^.

### Kaplan–Meier curves

Median abundance of each progressor correlated taxa was used to bin study participants into high or low abundance groups, independent of progressor status. Probability of disease progression over total disease duration in years stratified by low or high abundance of each taxa of interest was estimated using *survminer* version 0.4.9 and *survival* version 3.5–5 and plotted with *ggsurvfit* version 0.3.0^142–144^. To calculate hazard ratios and confidence intervals, cox regression analysis was performed using *finalfit* version 1.0.6^145^.

### Pathway analysis

To ascertain the inferred metagenomic signature associated specifically with progressor status or constipation, 16S data was subset to include only those ASVs associated with worsening disease or constipation by Spearman’s rank correlation filtered at rho ≥|0.2|, at p_adj_ ≤ 0.05 (Tables S8–S20) and analyzed with *PICRUSt2* to impute functional potential^54,55^. The resulting per sample unstratified enzyme commission (EC) numbers and MetaCyc pathways including description, were analyzed using DESeq2 for differential abundance testing^56^. ASV stratified matrices of EC numbers were also generated with *PICRUSt2* for mapping of per ASV contribution to enzymes and pathways. Differentially abundant pathways and ECs were imported to MetaCyc as SmartTables to visually represent overlap between pathways, ECs and contributing ASVs.

### Random-forest predictive modeling

Classifiers were generated using the *randomForest* package version 4.7–1.1^146^. All classifiers used a balanced bagging approach selecting 50% of samples from each class for each tree with class weight balanced by n_total_ / (2*n_class_) and leave one out cross-validation. The optimal number of variables sampled in each node split (mtry), size of terminal nodes (nodesize), and number of trees (ntree) were determined using the trainControl function in *caret* version 6.0–94 for algorithm tuning^147^. Classifiers incorporating clinical features as predictors exclude progressor status, progressor severity, final EDSS and sample during period given this variable is dependent on patient sex. Confusion matrices were plotted using the plot_rf_confusion tool from *modelmisc* version 0.1.1^148^. Model performance was evaluated as class probability using the predict function in *randomForest*. ROC curves were generated using *ROCR* version 1.0–11 and *pROC* version 1.58.0^149,150^.

### Supplementary Information

Supplementary Figures.

Supplementary Tables.

## Data Availability

All data that support the finding of this study generated and analyzed for the current study are available from the corresponding author upon request. The datasets generated and/or analyzed during the current study are available at the NCBI Sequence Read Archive repository, https://www.ncbi.nlm.nih.gov/sra (BioProject ID PRJNA1055510).
