# Supplementary material for: Identification of commensal gut microbiota signatures as predictors of clinical severity and disease progression in multiple sclerosis
Source: Sci Rep. 2024 Jul 3;14:15292. doi: 10.1038/s41598-024-64369-x (PMC11222390; doi:10.1038/s41598-024-64369-x)
Supplement: Supplementary file 1 — Supplementary Figures. [file 41598_2024_64369_MOESM1_ESM.docx]

**SUPPLEMENTAL FIGURES**
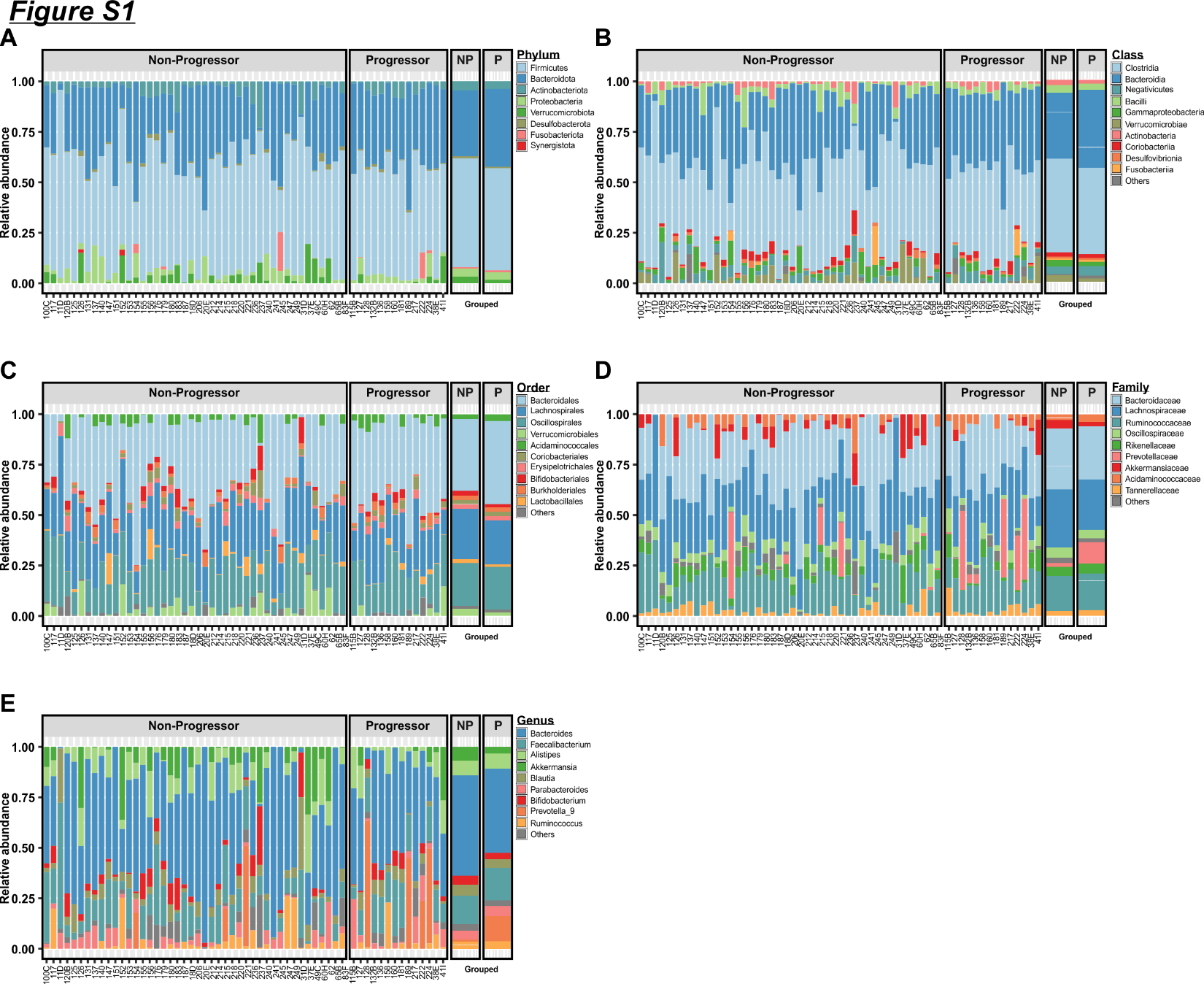
***Figure S1.* *Taxonomic distribution of gut microbiota in MS disease non-progressors and progressors.*** Extended data comparing the composition of gut microbiota in MS disease non-progressors and progressors. Distribution of top 10 most abundant (**A**) phyla (**B**) class (**C**) order (**D**) family and (**E**) genera. Per sample (left) and group (right) stacked bar plots at each taxonomic rank are represented as relative proportion of total 16S V4 amplicon reads.

***
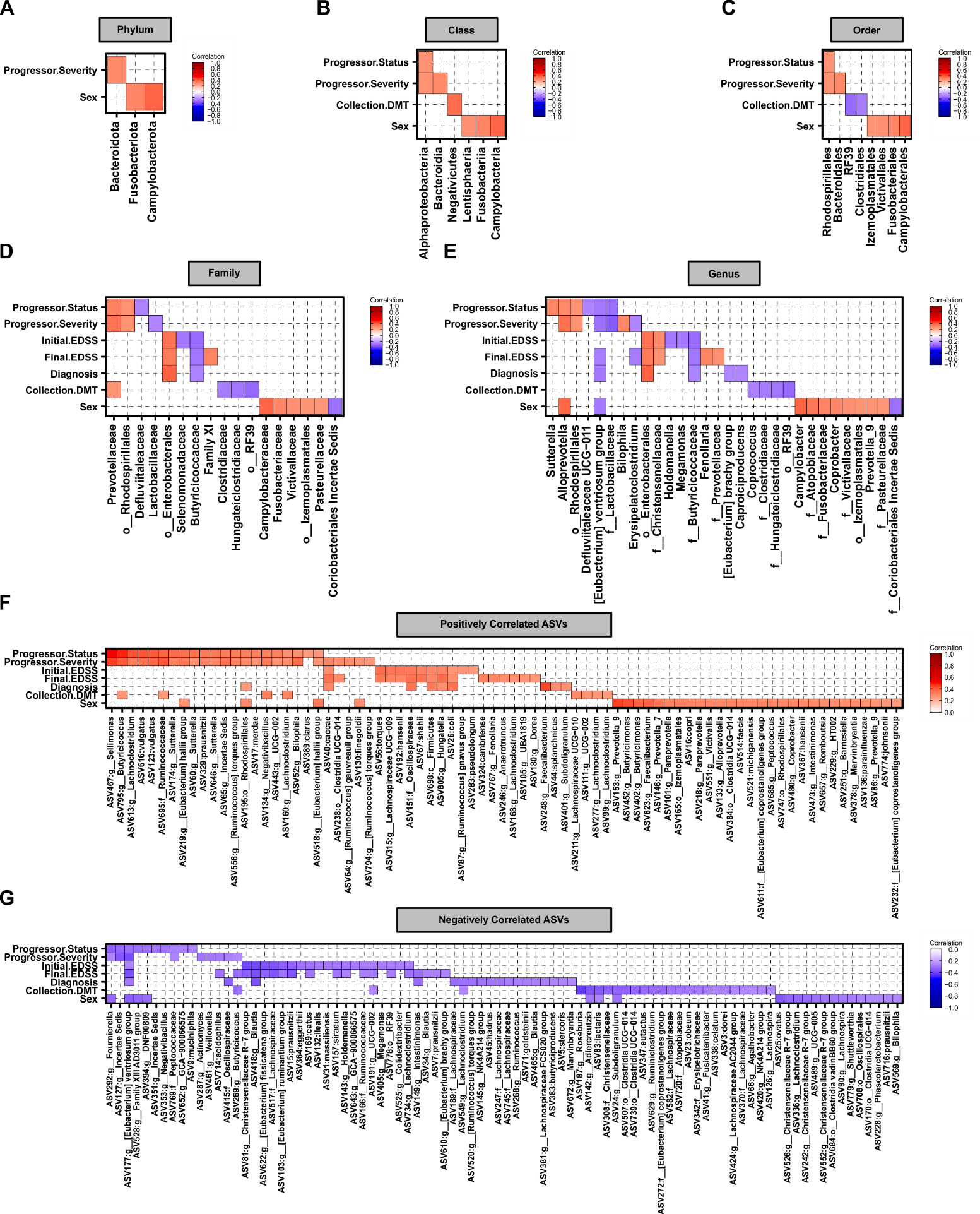
Figure S2. Association of gut microbiota taxa with progressor status and closely related subject metadata.*** Association of (**A**) phyla (**B**) class (**C**) order (**D**) family (**E**) genera and (**F**) positively or (**G**) negatively associated ASVs with progressor status and closely related subject metadata, as determined by Spearman rank correlation ≥|0.2|, at p_adj_ ≤ 0.05. Taxa are sorted from high to low rho-value within each metadata group top to bottom on y-axes, where warmer colors are indicative of positive association (increased abundance) and cooler colors represent negative association (decreased abundance).


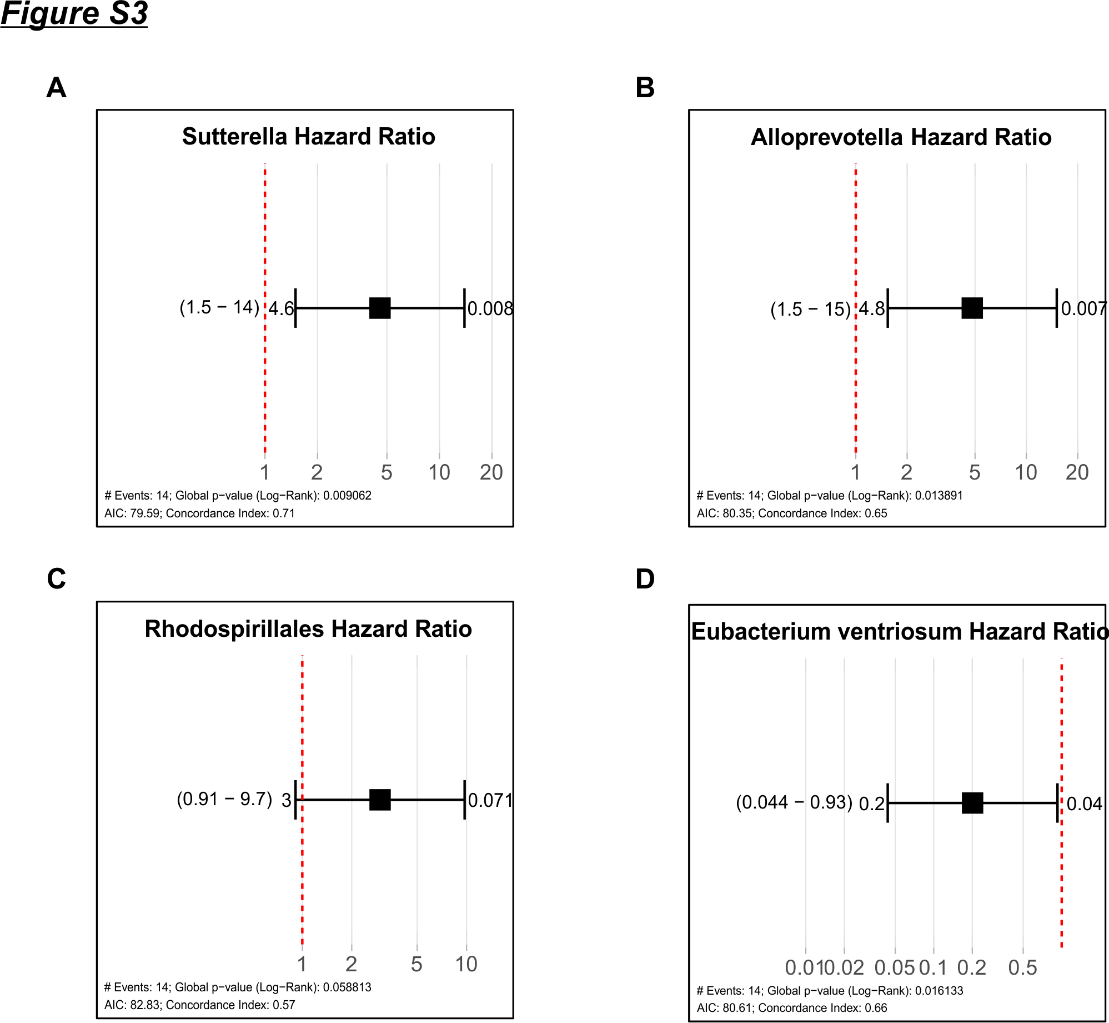
***Figure S3. Hazard ratio forest plots derived from cox proportional hazards modeling*.** Cox proportional hazards regression models were fit to predict hazard ratios for disease progression over subject disease duration (yrs) using cohorts stratified based on abundance of (**A**) *Sutterella* (low/high), (**B**) *Alloprevotella* (low/high), (**C**) *Rhodospirillales* (low/high), and (**D)** *Eubacterium ventriosum* (low/high). Forest plots depict hazard ratio confidence intervals with Wald test p-values shown right and global log-rank p-values bottom left.

***
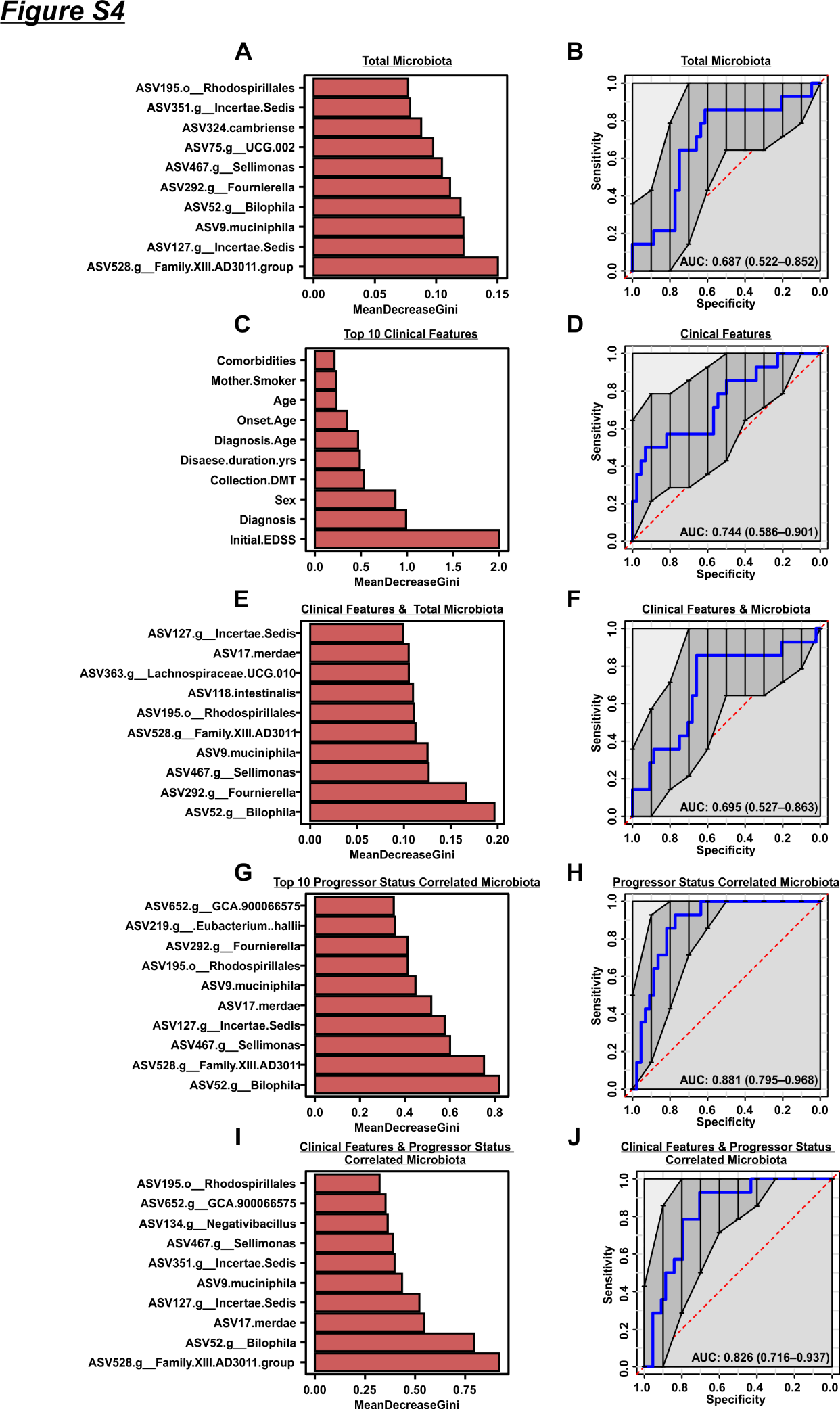
Figure S4. Random forest classifiers and receiver operating characteristic (ROC) curves for predicting disease progression.*** Top 10 variables of importance by mean decrease in Gini coefficient and ROC curve analysis are shown for Random Forest classifiers train on (**A**) and (**B**) total ASV level 16S abundance data, (**C**) and (**D**) patient metadata available at study baseline, (**E**) and (**F**) total ASV level 16S abundance data when combined with patient metadata, (**G**) and (**H**) only the ASVs correlated with progressor status as in **Fig. 4A**, and (**I**) and (**J**) ASVs correlated with progressor status with inclusion of patient baseline clinical metadata. All Random Forest classifiers were bootstrapped 1000 times using a balanced bagging approach with leave-one-out cross validation.


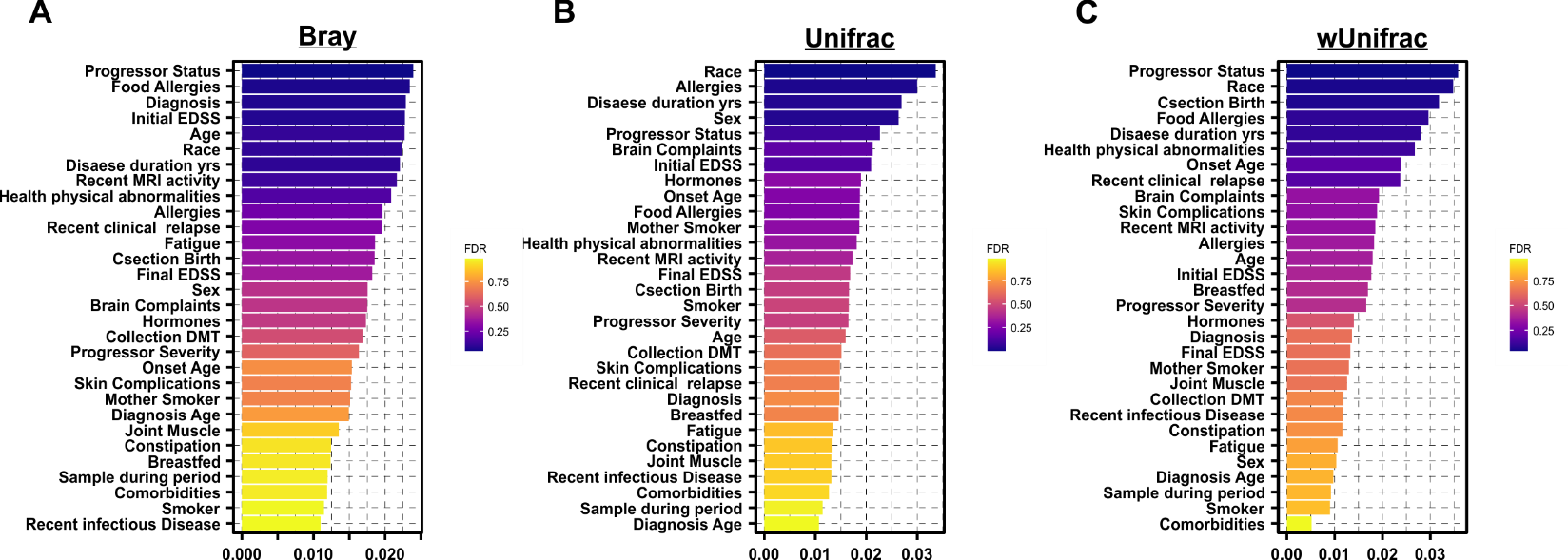


***Figure S5. Additional beta diversity analyses of study metadata*.**  PERMANOVA of (**A**) Bray-Curtis dissimilarity, (**B**) UniFrac distance, and (**C**) weighted UniFrac distance with the percent of variance explained by each metadata feature as R^2^ on x-axis and colored by FDR, as determined by the *adonis* test.

***
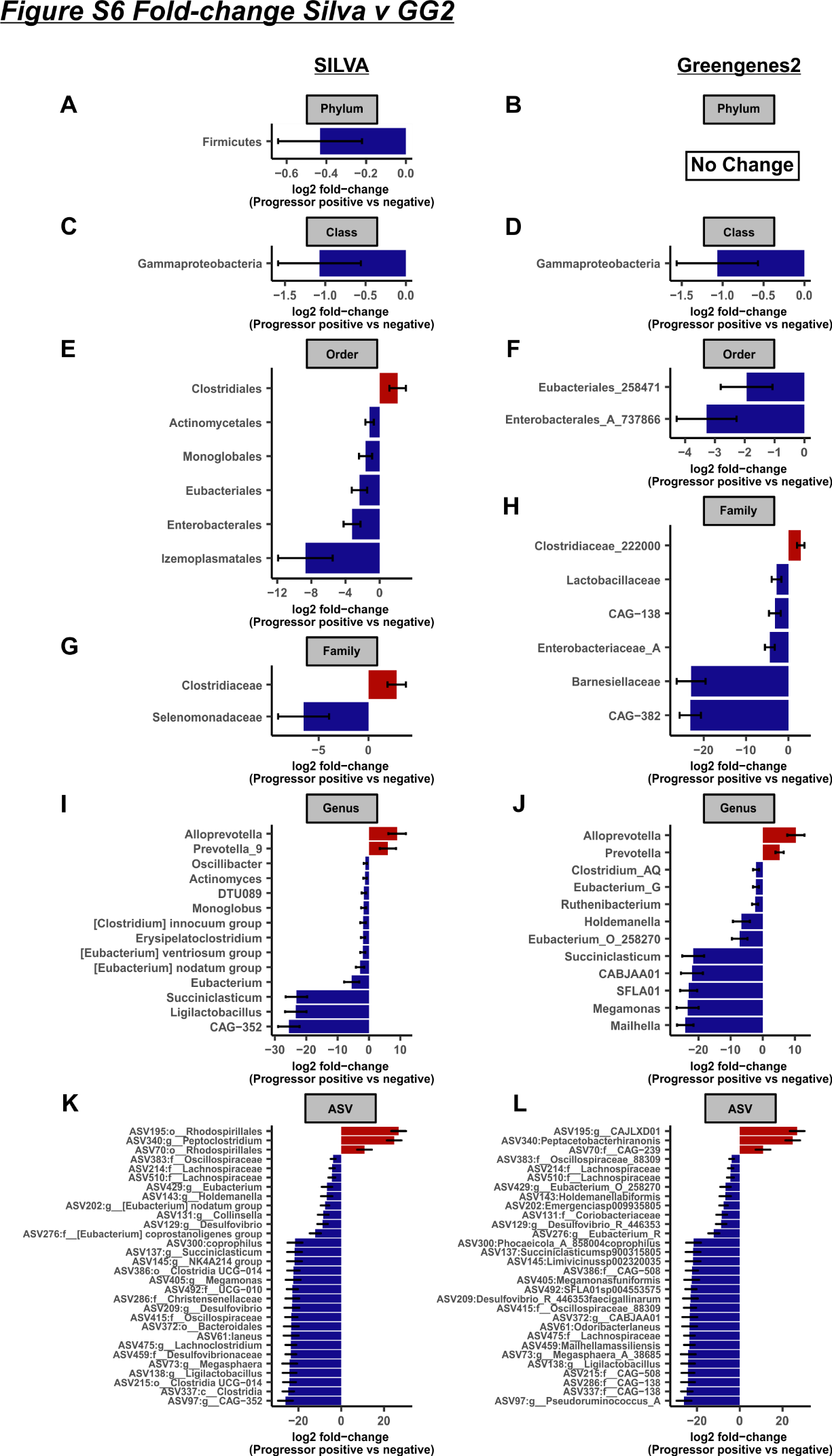
Figure S6. Concordance between SILVA and Greegenes2 taxonomic assignment*.**  Differentially abundant taxa between subjects with or without disease progression by (**A**) and (**B**) phylum, (**C**) and (**D**) class, (**E**) and (**F**) order, (**G**) and (**H**) family, (**I**) and (**J**) genus, and (**K**) and (**L**) top 30 ASVs represented as taxonomic best-hit, as determined by DESeq2 analysis, using a cutoff of p_adj_ ≤ 0.05. Taxonomy assigned using the *SILVA* database is show left with updated *Greengenes2* taxonomy shown right. Log2 fold-change reflects increased abundance in progressors when positive, and decreased abundance when negative.

***
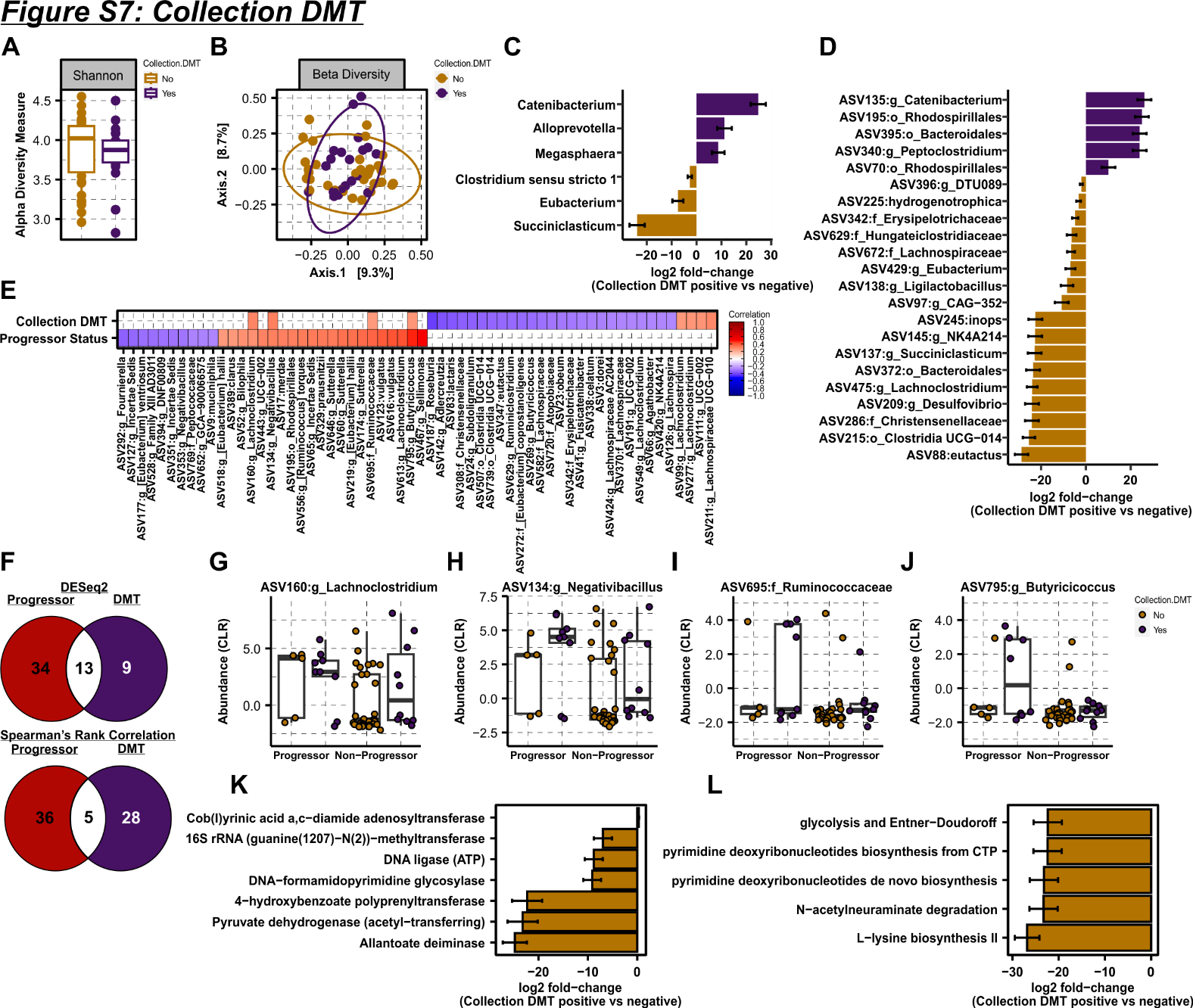
Figure S7. DMT usage is associated with both shared and unique microbial signatures compared to disease progression*.**  (**A**) Alpha (Shannon) and (**B**) beta (Bray-Curtis dissimilarity) diversity analysis in subjects utilizing or not DMTs at the time of fecal collection, as analyzed using Wilcoxon rank sum non-parametric or *adonis* tests, respectively. Differentially abundant taxa by (**C**) genus and (**D**) ASVs represented as taxonomic best-hit between subjects with or without collection DMT usage, as determined using DEseq2 using a cutoff of p_adj_ ≤ 0.05. Log2 fold-change reflects increased abundance in subjects with DMT usage when positive and decreased abundance when negative. (**E**) Association of ASVs with DMT usage and progressor status, as determined by Spearman rank correlation ≥|0.2|, at p_adj_ ≤ 0.05. ASVs are sorted from high to low rho-value within each metadata group top to bottom on y-axes, where warmer colors are indicative of positive association (increased abundance) and cooler colors represent negative association (decreased abundance). (**F**) Venn diagrams of shared or divergent ASVs between disease progressors and subjects utilizing DMTs as determined by DESeq2 (p_adj_ ≤ 0.05) and Spearman rank correlation ≥|0.2|, at p_adj_ ≤ 0.05. ASVs associated with both progressor status and DMT usage are plotted as CLR transformed abundance for shared ASVs in (**G-J**). (**K**) Differentially abundant enzymes and (**L**) pathways from the inferred metagenome of DMT associated ASVs. Total metagenomic potential was inferred with PICRUSt2 and differential abundance analyzed for the subset of ASVs associated with constipation using DESeq2 at p_adj_ ≤ 0.05.

***
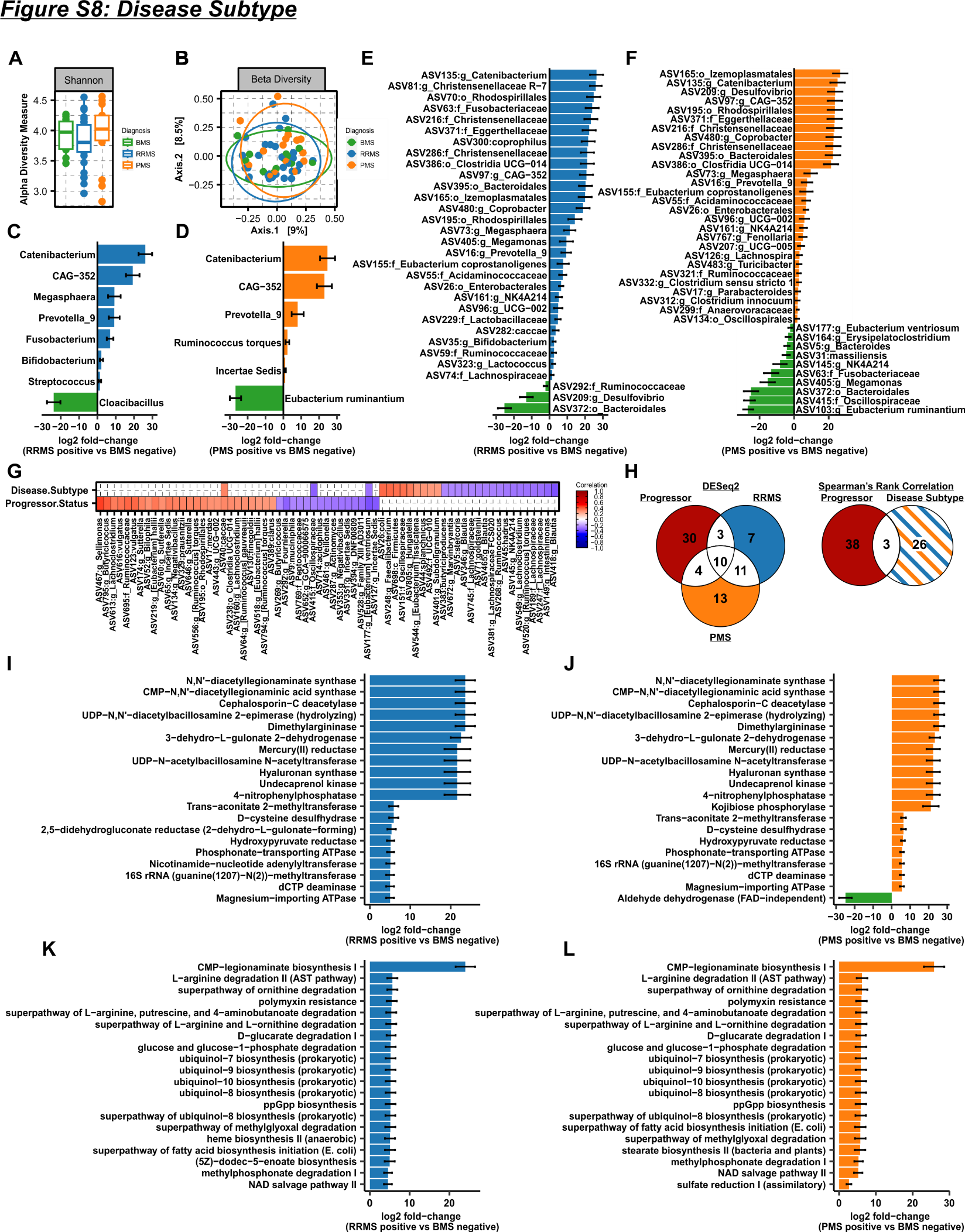
***

***Figure S8. Comparison of disease subtype and progressor status associated microbial signatures*.**  Disease subtype was binned as BMS=0 (control), RRMS=1 (inflammatory mediated) and PPMS/RRMS (PMS)=2 (non-inflammatory mediated) for comparison to disease progression status. (**A**) Alpha (Shannon) and (**B**) beta (Bray-Curtis dissimilarity) diversity analysis of MS disease subtype, as analyzed using Wilcoxon rank sum non-parametric or *adonis* tests, respectively. Differentially abundant taxa comparing genera (**C**) BMS vs. RRMS or (**D**) BMS vs. PMS and comparing ASVs represented as taxonomic best-hit in (**E**) BMS vs. RRMS or (**F**) BMS vs. PMS, as determined using DEseq2 using a cutoff of p_adj_ ≤ 0.05. Log2 fold-change reflects increased abundance in subjects with RRMS or PMS compared to BMS when positive and decreased abundance when negative. (**G**) Association of ASVs with MS subtype and progressor status, as determined by Spearman rank correlation ≥|0.2|, at p_adj_ ≤ 0.05. ASVs are sorted from high to low rho-value within each metadata group top to bottom on y-axes, where warmer colors are indicative of positive association (increased abundance) and cooler colors represent negative association (decreased abundance). (**H**) Venn diagrams of shared or divergent ASVs between disease progressors and MS subtype as determined by DESeq2 (p_adj_ ≤ 0.05) and Spearman rank correlation ≥|0.2|, at p_adj_ ≤ 0.05. Differentially abundant enzymes comparing (**I**) BMS vs. RRMS or (**J**) BMS vs PMS and pathways comparing (**K**) BMS vs. RRMS or (**L**) BMS vs PMS from the inferred metagenome of MS subtype associated ASVs. Total metagenomic potential was inferred with PICRUSt2 and differential abundance analyzed for the subset of ASVs associated with MS-subtype using DESeq2 at p_adj_ ≤ 0.05.


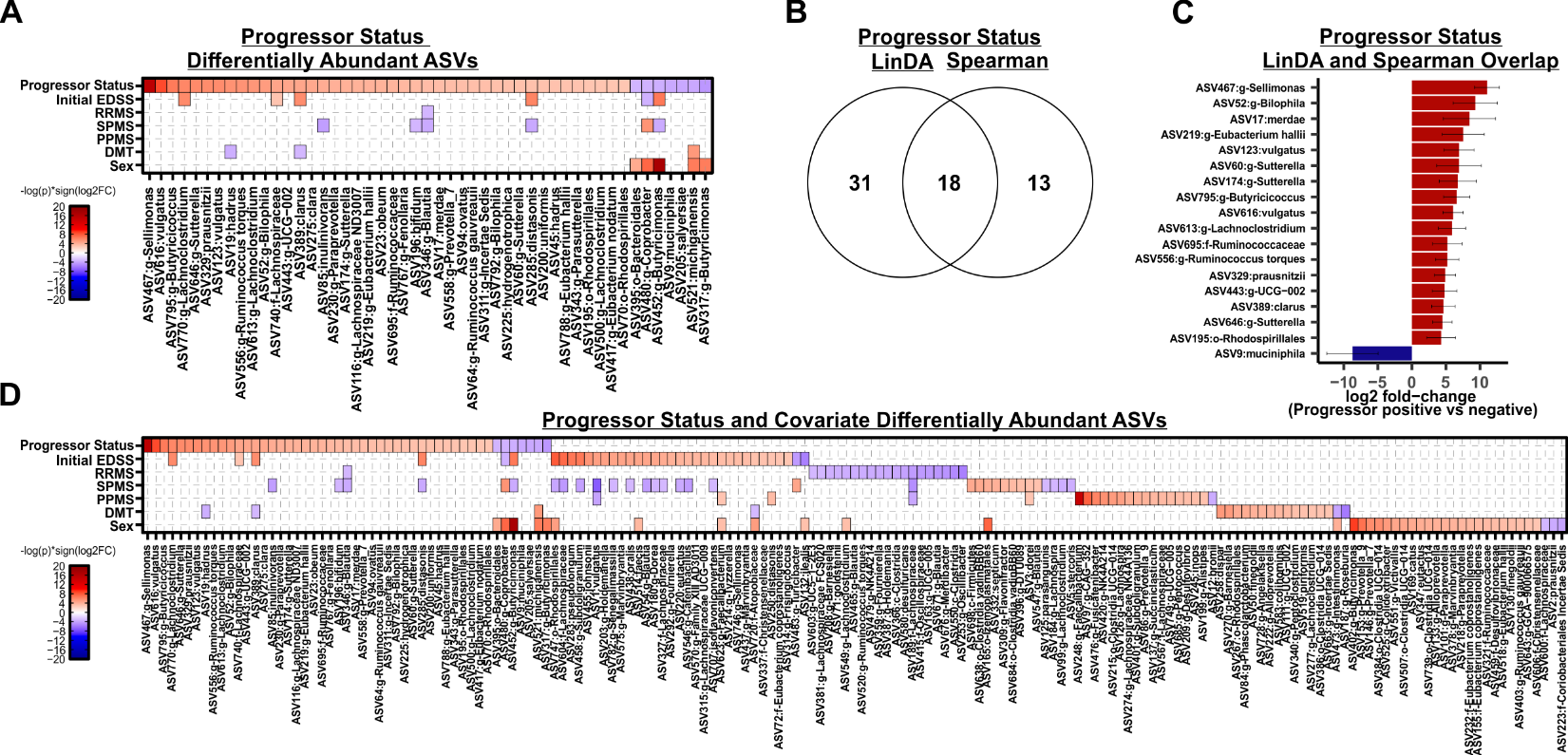


***Figure S9. Mixed linear modeling to adjust for confounders of disease progressor differentially abundant ASVs*.**  Mixed linear modeling to adjust for initial EDSS, diagnosis, DMT, and sex was conducted using LinDA. Differentially abundant ASVs associated with disease progression (**A**) at p≤0.05 are compared to results obtained using Spearman rank correlation at ≥|0.2|, padj ≤ 0.05 in (**B**) with shared results graphed as log2 fold-change reflecting increased abundance in progressors when positive, and decreased abundance when negative in (**C**). Complete results obtained using LinDA for progressor status and confounders are shown in (**D**).
